# Is body mass index associated with outcomes of mechanically ventilated adult patients in intensive critical units? A systematic review and meta-analysis

**DOI:** 10.1371/journal.pone.0198669

**Published:** 2018-06-08

**Authors:** Yonghua Zhao, Zhiqiang Li, Tao Yang, Meiping Wang, Xiuming Xi

**Affiliations:** 1 Department of Critical Care Medicine, Fuxing Hospital, Capital Medical University, Beijing, China; 2 Department of Critical Care Medicine, Langfang People’s Hospital, Langfang, China; 3 Department of Critical Care Medicine, Hebei United University Affiliated Hospital, Tangshan, China; Azienda Ospedaliero Universitaria Careggi, ITALY

## Abstract

**Background:**

Obesity paradox refers to lower mortality in subjects with higher body mass index (BMI), and has been documented under a variety of condition. However, whether obesity paradox exists in adults requiring mechanical ventilation in intensive critical units (ICU) remains controversial.

**Methods:**

MEDLINE, EMBASE, China Biology Medicine disc (CBM) and CINAHL electronic databases were searched from the earliest available date to July 2017, using the following search terms: “body weight”, “body mass index”, “overweight” or “obesity” and “ventilator”, “mechanically ventilated”, “mechanical ventilation”, without language restriction. Subjects were divided into the following categories based on BMI (kg/m^2^): underweight, < 18.5 kg/m^2^; normal, 18.5–24.9 kg/m^2^; overweight, BMI 25–29.9 kg/m^2^; obese, 30–39.9 kg/m^2^; and severely obese > 40 kg/m^2^. The primary outcome was mortality, and included ICU mortality, hospital mortality, short-term mortality (<6 months), and long-term mortality (6 months or beyond). Secondary outcomes included duration of mechanical ventilation, length of stay (LOS) in ICU and hospital. A random-effects model was used for data analyses. Risk of bias was assessed using the Newcastle-Ottawa quality assessment scale.

**Results:**

A total of 15,729 articles were screened. The final analysis included 23 articles (199,421 subjects). In comparison to non-obese patients, obese patients had lower ICU mortality (odds ratio (OR) 0.88, 95% CI 0.0.84–0.92, I^2^ = 0%), hospital mortality (OR 0.83, 95% CI 0.74–0.93, I^2^ = 52%), short-term mortality (OR 0.81, 95% CI 0.74–0.88, I^2^ = 0%) as well as long-term mortality (OR 0.69, 95% CI 0.60–0.79, I^2^ = 0%). In comparison to subjects with normal BMI, obese patients had lower ICU mortality (OR 0.88, 95% CI 0.82–0.93, I^2^ = 5%). Hospital mortality was lower in severely obese and obese subjects (OR 0.71, 95% CI 0.53–0.94, I^2^ = 74%, and OR 0.80, 95% CI 0.73–0.89, I^2^ = 30%). Short-term mortality was lower in overweight and obese subjects (OR 0.82, 95% CI 0.75–0.90, I^2^ = 0%, and, OR 0.75, 95% CI 0.66–0.84, I^2^ = 8%, respectively). Long-term mortality was lower in severely obese, obese and overweight subjects (OR 0.39, 95% CI 0.18–0.83, and OR 0.63, 95% CI 0.46–0.86, I^2^ = 56%, and OR 0.66, 95% CI 0.57–0.77, I^2^ = 0%). All 4 mortality measures were higher in underweight subjects than in subjects with normal BMI. Obese subjects had significantly longer duration on mechanical ventilation than non-obese group (mean difference (MD) 0.48, 95% CI 0.16–0.80, I^2^ = 37%), In comparison to subjects with normal BMI, severely obese BMI had significantly longer time in mechanical ventilation (MD 1.10, 95% CI 0.38–1.83, I^2^ = 47%). Hospital LOS did not differ between obese and non-obese patients (MD 0.05, 95% CI -0.52 to 0.50, I^2^ = 80%). Obese patients had longer ICU LOS than non-obese patients (MD 0.38, 95% CI 0.17–0.59, I^2^ = 70%). Hospital LOS and ICU LOS did not differ significantly in subjects with different BMI status.

**Conclusions:**

In ICU patients receiving mechanical ventilation, higher BMI is associated with lower mortality and longer duration on mechanical ventilation.

## Introduction

Obesity, typically defined as BMI of ≥30 kg/m^2^, is an increasing public concern [1]. It is one of the top 10 risk factors of chronic diseases [2–4]. Nearly 300,000 Americans die from a range of diseases related to obesity each year, and the economic burden exceeds more than 5% of the national health expenditure [5, 6]. Consistently with the trend in the general population, the number of obese patients admitted to ICUs is rapidly rising [7].

Obesity has been associated with increasing mortality in critically ill patients [8–10]. A variety of factors contribute to the association between obesity and mortality, including a series of physiological changes that result in poor stresses related to acute inflammatory and immune responses, or in many comorbidities including diabetes, cardiovascular events, respiratory diseases and cancer [11]. However, “obesity paradox” (namely, lower mortality in obese subjects) has also been reported in ICU patients in other studies [12–13]. The relationship between obesity and mortality of ICU patients thus remain largely unclear[14]. In the current study, we conducted a systematic review and meta-analysis of published studies to investigate the relationship between BMI and ICU outcomes in patients received mechanical ventilation.

## Materials and methods

### Literature search and study selection

We conducted a comprehensive electronic search of MEDLINE, EMBASE, CBM and Cochrane Central Register of Controlled Trials databases (CENTRAL) from the earliest available date to July 2017. The search strategy is described in S1 Text. Two authors (Yonghua Zhao, Zhiqiang Li) manually searched the references listed in each identified article and other relevant articles to identify all eligible studies. The search was not restricted in publication type or languages.

### Inclusion and exclusion criteria

For inclusion in data analysis, studies must meet all following criteria: 1) cohort studies in patients receiving mechanical ventilation in ICU; 2) patients across two or more BMI categories; 3) outcomes include all-cause mortality, including ICU mortality, long-term mortality, short-term mortality, hospital mortality, or duration of mechanical ventilation, hospital and ICU LOS. The exclusion criteria included: patients under 18 years of age, review articles, case reports or animal experiment.

### Data extraction and outcomes

Data extraction was carried out as recommended by the *Cochrane* handbook, and included authors, year of publication, study design, participants, BMI categories, demographic characteristics, severity of illness, measurement of BMI. BMI was classified into 5 categories using the National Institutes of Health (NIH) criteria [15]: 1) underweight: BMI <18.5 kg/m^2^; 2) normal weight: BMI 18.5–24.9 kg/m^2^; 3) overweight: BMI 25–29.9 kg/m^2^; 4) obese: BMI 30–39.9 kg/m^2^; 5) severely obese: BMI ≥40 kg/m^2^. Both review of full texts and extraction of data were independently performed by two reviewers (Yonghua Zhao, Zhiqiang Li). Duplicate reports were discarded by screening titles and abstracts. Any disagreement between the two primary reviewers was resolved by discussion with the third party (Xiuming Xi).

### Quality assessment

Risk of bias of individual studies at the outcome level was assessed using the Newcastle-Ottawa quality assessment scale: 4 for selection, 2 for comparability and 3 for outcome. Study quality was rated based on the score: 1 (very poor) to 9 (very high) (S1 Table).

### Statistical analysis

Statistical analysis was performed using the Cochrane systematic review software Review Manager (RevMan; Version 5.3.5). Data analysis was performed using a random-effects model developed by DerSimonian and Laird [16]. Dichotomous variables are presented as odds ratio (OR) with 95% confidence intervals (CI). Continuous variables are presented as mean difference (MD) with 95% confidence intervals. Significant heterogeneity was defined as P < 0.1 in χ2 test and I^2^ > 50%. If the literature reported the median or range of continuous variables, we used the median as the mean and the method provided by the Cochrane handbook [17] to calculate the standard deviation, and carried out sensitivity analysis. Publication bias was assessed with a funnel plot when there were 10 or more eligible studies.

## Results

**Fig 1:**
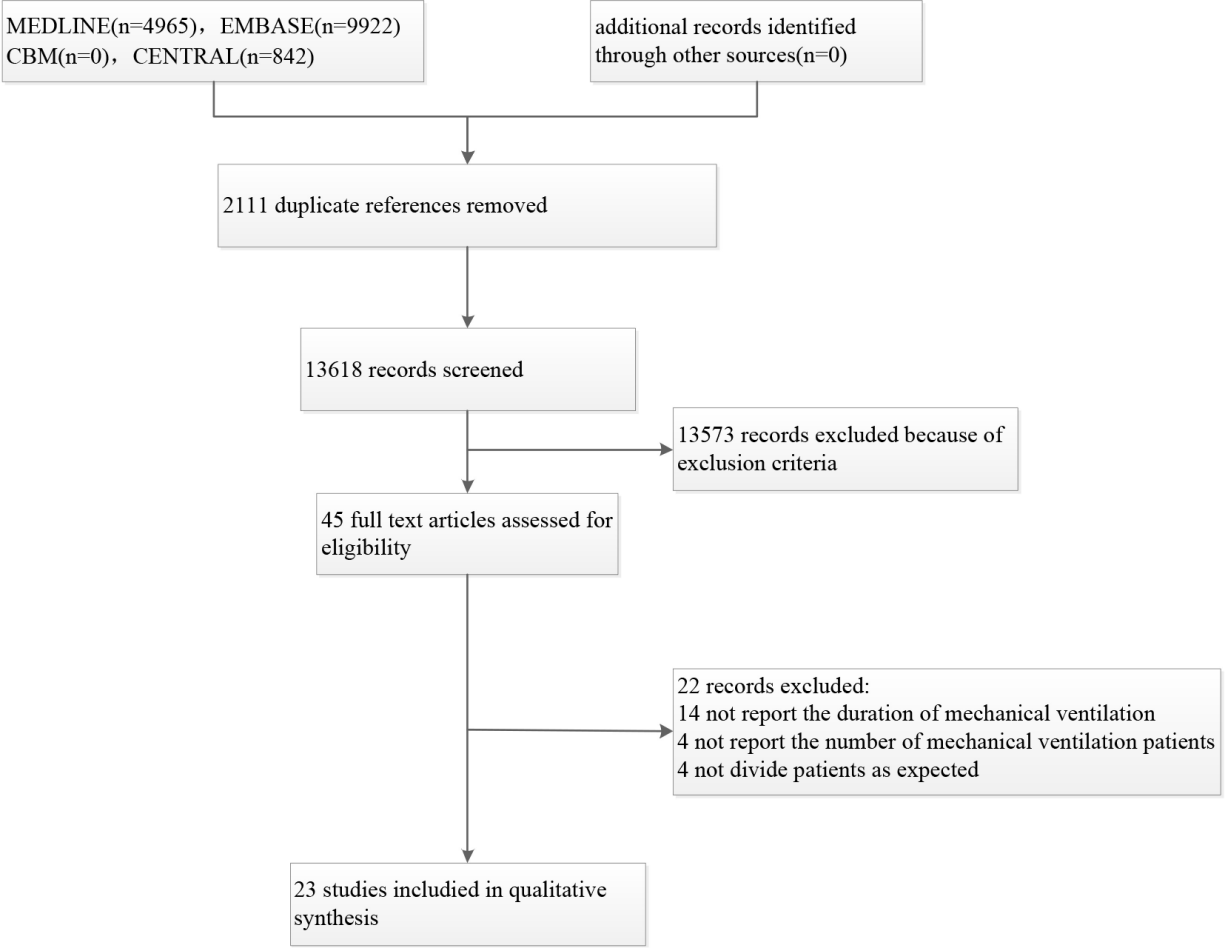
Flow diagram of study selection process.

The search identified a total of 15,729 articles. After screening the titles and abstracts and removal of duplicates, 23 articles (199, 421 subjects) were included in the meta-analysis (Fig 1).

### Study description

All eligible studies were approved by their corresponding institutional ethics committee. A total of 199,421 subjects participated in the 23 studies, which consisted of 14 prospective [8, 13, 18–29] and 9 retrospective cohort studies [9–12, 30–35]. Fourteen studies were from North America [9, 10, 12, 13, 19, 20, 21, 25, 27, 28, 30, 32–34], one from South America [31], five from Europe [8, 18, 22, 23, 24, 29], and two from Australia [26, 35]. In seven studies [8, 9, 18, 19, 22, 31, 32], BMIs was classified only into obese versus non-obese (Table 1). Five studies reported short-term mortality [20, 23, 26, 28, 30]. Four studies reported long-term mortality [14, 19, 26, 28].

**Table 1:** Study characteristics by body mass index category.

References	Country	Study design	Study period	ICU type	BMI categories(kg/m^2)^	Sample size	Age	Male%	Severity of illness
							Mean ± SD or median		
									Mean ± SD or median
Wardell S[19]	Canada	Prospective	Jan 2008 to Mar 2009	Mixed ICU					APACHE II
					Nonobese: < 30.0	202	51.8(18.9)	34.7	20.8(8.5)
					Obese: ≥ 30.0	146	57.7(16.4)	42.5	23.5(8.1)
Lee CK[30]	USA	Retrospective	Jan to Dec 2009	Medical ICU					APACHE II
					Nonobese: < 30.0	306	63.0(16.5)	48.4	24.7(8.1)
					obese: ≥ 30.0	85	59.6(16.2)	59.6	23.4(8.0)
					severely obese: > 35.0	113	NR	NR	NR
O'Brien JM[13]	USA	Prospective	Feb 2006 to Jan 2008	Medical ICU					NR
					Normal: < 25.0	213	58.3(17.1)	56.8	
					Overweight: 25.0 to 30.0	129	57.2(15.5)	54.3	
					Obese: ≥ 30.0	238	56.2(13.8)	52.9	
Martino JL[20]	33 countries	Prospective	2007 to 2009	ICU		`			APACHE II
					Normal:18.5 to 24.9	3490	58.6(18.9)	61	22.2(8.0)
					Overweight:25 to 29.9	2604	60.2(17.4)	66.4	22.4(8.1)
					Obese: 30 to 39.9	1772	60.8(15.2)	57.7	22.9(8.0)
					Extremely obese: ≥ 40.0	524	NR	44.8	NR
Anzueto A[21]	USA	Prospective	Apr 2004	ICU					SAPS II
					Underweight: < 18.5	184	55(19)	54.4	43(19)
					Normal: 18.5 to 24.9	1995	57(19)	58.8	43(18)
					Overweight: 25.0 to 29.9	1781	61(17)	67.1	43(17)
					Obese: 30 to 39.9	792	61(14)	55	44(17)
					Severely obese: > 40.0	216	55(14)	45.9	42(17)
Diaz E[22]	Spain	Prospective	Jan 2010	ICU					APACHE II
					Nonobese:< 30.0	265	43(15.4)	59.6	13.3(7.4)
					Obese: ≥ 30.0	150	43.9(12.3)	55.3	13.5(6.5)
Moock M[31]	Brazil	Retrospective	Apr 2005 to Nov 2008	ICU					APACHE II
					Nonobese:< 30.0	146	49.1 (57.5–69.6)	72	8 (12–16)
					Obese: ≥ 30.0	73	49.7 (59.4–69.7)	33	8 (16–20)
Morris AE[25]	USA	Prospective	Apr 1999 to Jul 2000	ICU					APACHE II
					Underweight : < 18.5	50	64.7(18.4)	56	82.3(31.5)
					Normal: 18.5 to 24.9	301	61.5(18.1)	64.8	74.9(29.2)
					Overweight: 25.0 to 34.9	237	58.9(17.4)	66.2	74.9(30.0)
					Obese : 35.0 to 39.9	183	57.0(15.9)	63.4	70.3(29.8)
					Severely obese: ≥ 40.0	54	54.7(13.9)	48.1	75.0(35.1)
Peake SL[26]	Australia	Prospective	2001	ICU					APACHE II
					Underweight : < 18.5	24	62.0(16.5)	58.3	20.8(8.0)
					Normal: 18.5 to 24.9	129	61.3(20.4)	60.5	19.9(7.9)
					Overweight: 25.0 to 29.9	151	64.1(16.1)	58.3	19.9(7.9)
					Obese : 30.0 to 34.9	75	62.9(14.4)	57.3	19.9(8.7)
					Severely obese: ≥ 35.0	54	61.0(15.7)	50	19.4(8.0)
Brown CVR[9]	USA	Retrospective	1998 to 2003	ICU					ISS
					Nonobese:< 30.0	870	45(20)	71	21(12)
					Obese: ≥ 30.0	283	46(18)	70	21(13)
Ray DE[27]	USA	Prospective	Jan 1997 to Aug 2001	Medical ICU					APACHE II
					Underweight: < 20.0	350	62.2(20.2)	47.4	18.2(8.3)
					Normal:20.0 to 24.9	663	65.2(18.6)	54.6	18.4(8.9)
					Overweight:25.0 to 29.9	585	64.8(16.3)	54	18.3(9.3)
					Obese:30 to 39.9	396	61.7(16.7)	46.2	17.0(8.7)
					Severely obese: ≥ 40.0	154	57.4(16.0)	34.4	18.2(9.0)
Goulenok C[8]	France	Prospective	Jan 1999 to Jan 2000	Medical ICU					APACHE II
					Nonobese: < 27.0	598	48 (34–65)	41	32 (19–48)
					Obese: ≥ 27.0	215	58 (47–71)	42	36 (27–56)
El-Solh A [10]	USA	retrospective	January 1994 to June 2000	Medical and surgical ICU					APACHE II
					nonobese: < 30.0	132	46.2(21.7)	55.3	20.6(12.2)
					Morbidly obese: ≥ 40.0	117	44.4(18.2)	43.59	19.1(7.6)
Frat J[24]	France	Prospective	Sep 2002 to Jun 2004	ICU					SAPS II
					Nonobese: < 30.0	124	65(11)	64.52	45(14)
					Severely obese: ≥ 35.0	82	64(11)	59.76	45(16)
Alberda C[23]	37 countries	Prospective	Jan 2007	ICU					APACHE II
					Underweight: < 20.0	289	58.7(19.0)	50.9	22.04(7.71)
					Normal: 20.0 to 25.0	937	58.3(19.0)	59.8	21.40(8.15)
					Overweight: 25 to 30.0	818	60.0(17.8)	65.6	21.43(7.96)
					Obese: 30.0 to 35.0	395	62.2(15.3)	57.5	22.41(7.40)
					Severely obese: 35.0 to 40.0	162	62.3(13.7)	50.6	21.62(8.36)
					Morbidly obese: ≥ 40.0	171	56.9(13.3)	45	22.21(8.53)
Duane TM[32]	USA	Retrospective	Jan 2004 to Dec 2005	ICU	Nonobese: < 30.0	51	NR	NR	NR
					Obese: > 30.0	10			
O'Brien JM Jr[33]	USA	Retrospective	Dec 1995 to Sep 2001	ICU					NR
					Underweight: < 18.5	88	62.4(16.2)	46.6	
					Normal: 18.5 to 24.9	544	61.0(17.8)	56.4	
					Overweight: 25.0 to 29.9	399	59.4(16.7)	55.9	
					Obese : 30.0 to 39.9	326	58.0(16.3)	46.6	
					severely obese: ≥ 40.0	131	53.6(14.9)	33.6	
Tafelski S[18]	Germany	Prospective	Aug 2009 to Apr 2010	Surgical ICU					SAPS II
					Nonobese: < 30.0	451	63 (50–72)	57	36 (24–48)
					Obese: ≥ 30.0	130	64 (53–72)	53	35 (25–53)
Dennis, DM[35]	Australia	Retrospective	Nov 2012 to Jun 2014	ICU					APACHE II
					Underweight : < 18.5	18	56(39)	NR	19(12.0)
					Normal: 18.5 to 24.9	200	57(24)		18(10.0)
					Overweight: 25.0 to 29.9	249	55(25)		18(10.0)
					Obese : 30.0 to 39.9	216	60(19)		18(9.0)
					severely obese: ≥ 40.0	52	60(15)		19(9.5)
Lewis O[34]	USA	Retrospective	2012	Medical ICU					mortality prediction model II
					Underweight : < 18.5	61	59.62(18.73)	62.3	37.19(27.80)
					Normal: 18.5 to 24.9	206	58.06(16.08)	59.22	39.61(28.54)
					Overweight: 25.0 to 29.9	127	58.57(16.74)	49.61	36.28(28.76)
					Obese(class1) : 30.0 to 34.9	90	59.48(15.72)	41.11	33.04(27.7)
					Obese(class2): 35 to 39.9	57	59.07(16.51)	28.07	38.21(29359)
					severely obese: ≥ 40.0	64	54.64(17.41)	29.69	34.09(27.63)
Trivedi V[12]	USA	Retrospective	Jan 2010 to May 2011	Medical ICU					APACHE Ⅱ
					Underweight : < 18.5	41	61.8(18.2)	56.1	20.3(9.7)
					Normal: 18.5 to 24.9	259	61.3(19.8)	59.1	18.7(8.3)
					Overweight: 25.0 to 29.9	194	60.4(17.3)	59.3	18.0(8.9)
					Obese : 30.0 to 39.9	205	61.3(15.3)	44.9	18.7(9.6)
					severely obese: ≥ 40.0	59	NR	NR	NR
Pickkers P[29]	Dutch	Prospective	January 1,1999, to January 1,2010	ICU					SAPS II
					Underweight : < 18.5	5343	60.0 (46–74)	38.6	36.4(19.6)
					Normal: 18.5 to 24.9	74883	65.0 (50–75)	58.6	35.1 (19.2)
					Overweight: 25.0 to 29.9	52141	67.0 (55–75)	62.5	35.7(19.4)
					Obese(class1) : 30.0 to 34.9	14660	65.0 (54–74)	53.1	34.7(19.1)
					Obese(class2): 35 to 39.9	4339	62.0(51–71)	42.4	34.6(19.9)
					severely obese: ≥ 40.0	2992	57.0 (45–67)	34.6	31.6(20.8)
Abhyankar S[28]	USA	Prospective	from 2001 to 2008	Mixed ICU					SAPS
					Underweight : < 18.5	786	70.6(53.0–81.8)	56.4	12.3 (5.3)
					Normal: 18.5 to 24.9	5463	69.4(51.7–80.4)	45.2	12.2 (5.4)
					Overweight: 25.0 to 29.9	5276	67.2(53.6–77.8)	36	12.0 (5.3)
					Obese: ≥ 30.0	5287	62.3(51.7–3.2)	44.6	12.0 (5.3)

**BMI** body mass index; **ICU** intensive care unit; **NR** no report; **SAPSⅡ** simplified acute physiology score II; **APS** acute physiology score; **APACHEⅡ** Acute Physiology and Chronic Health Evaluation II; **ISS** Injury Severity Score.

### Publication bias

Since there were more than ten studies comparing the obese patients, it was possible to assess for publication bias, Funnel plot did not reveal significant publication bias on duration of mechanical ventilation (S1A Fig), ICU and hospital LOS (S1B and S1C Fig).

### Mortality

**Fig 2:**
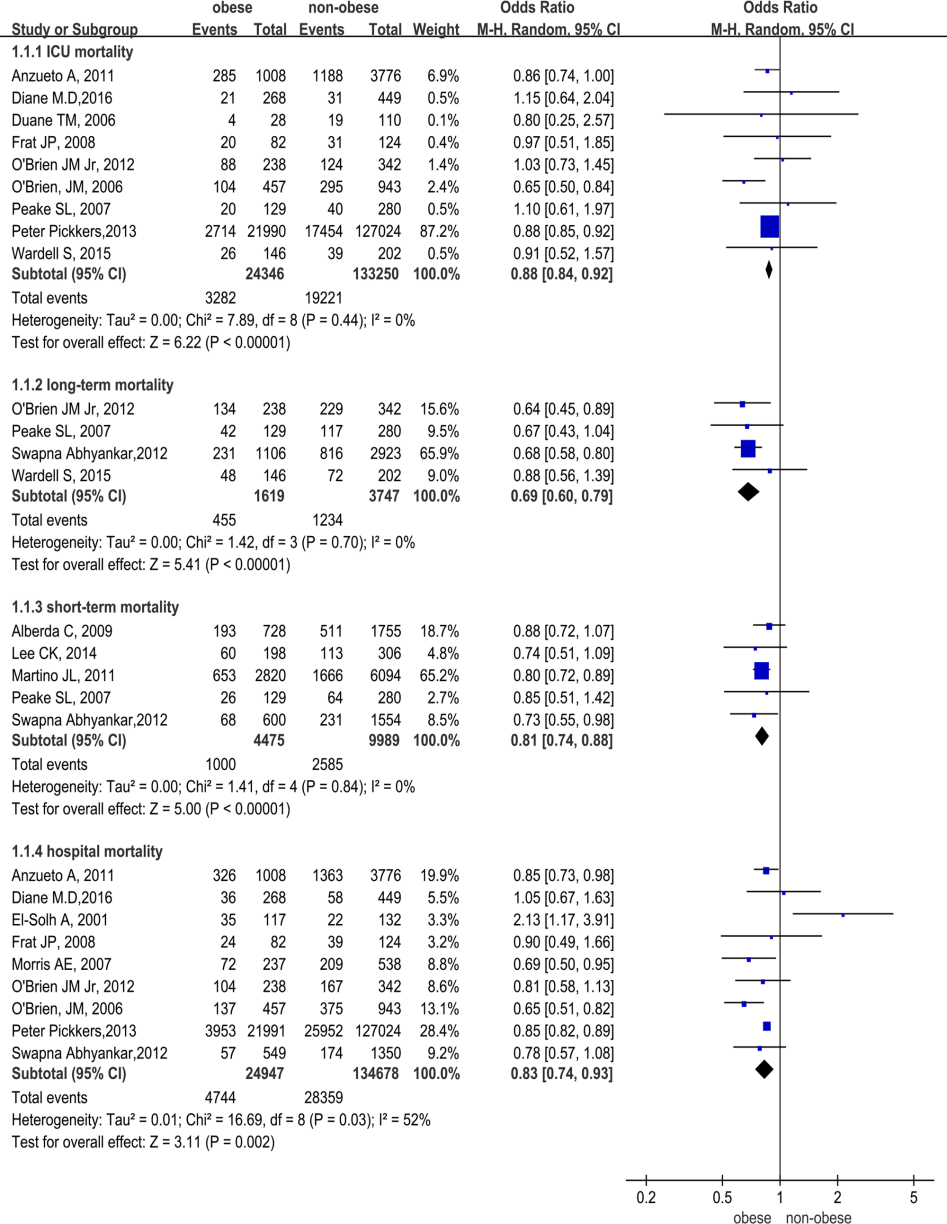
Obese and non-obese patients mortality.

**Fig 3:**
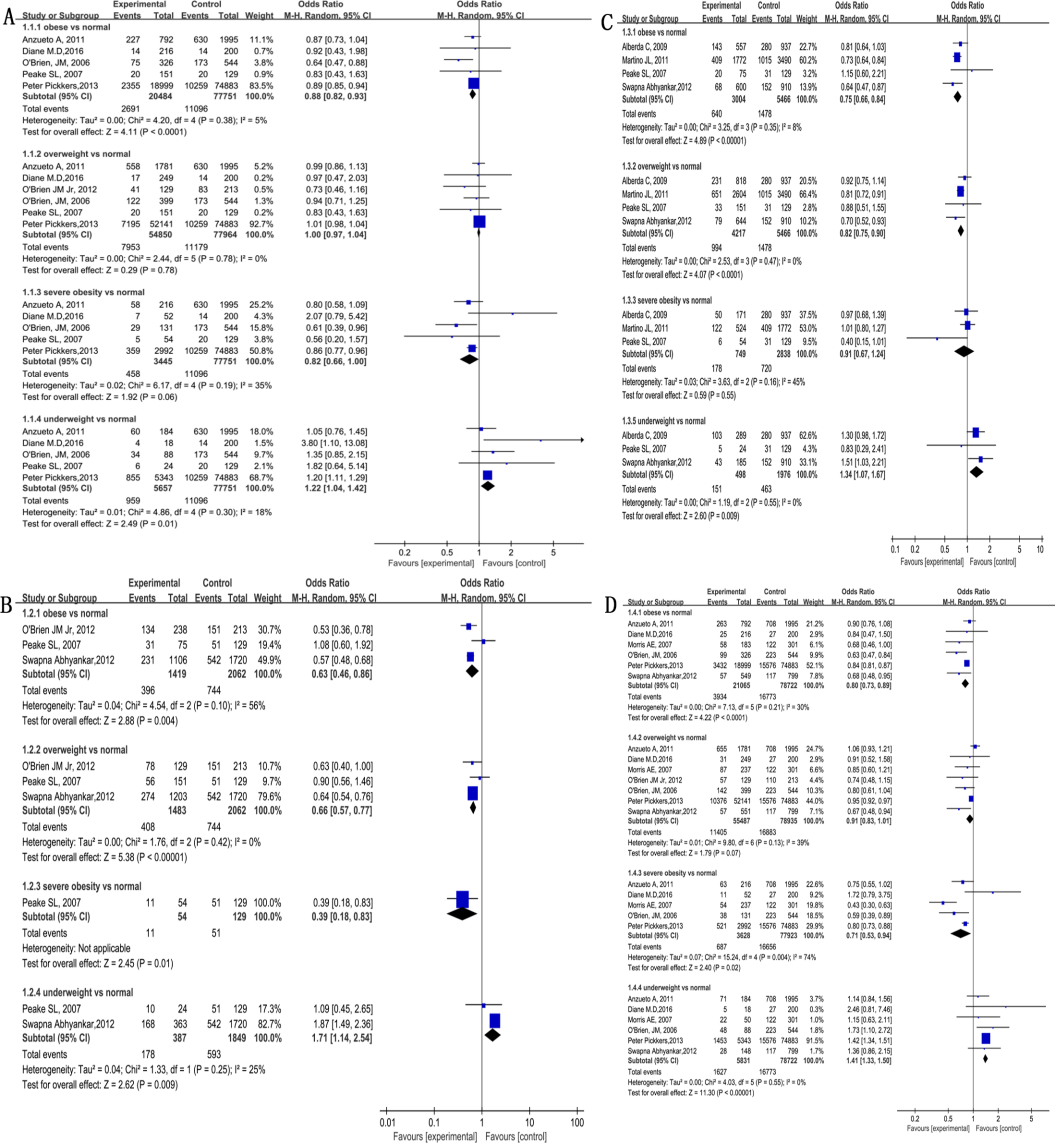
Association of obesity and mortality in in mechanical ventilation patients in ICU. (A) ICU mortality. (B) long-term mortality. (C) short-term mortality. (D) hospital mortality.

In comparison to non-obese subjects, obese patients had lower ICU mortality (OR 0.88, 95% CI 0.84–0.92, Z = 6.22, P<0.00001 I^2^ = 0%), hospital mortality (OR 0.83, 95% CI 0.74–0.93, Z = 3.11, P<0.002, I^2^ = 52%), short-term mortality (OR 0.81, 95% CI 0.74–0.88, Z = 5.00, P<0.00001, I^2^ = 0%), as well as long-term mortality (OR 0.69, 95% CI 0.60–0.79, Z = 5.41, P<0.00001, I^2^ = 0%, Fig 2). In comparison to subjects with normal BMI, The results are as follows: obese patients had lower ICU mortality (OR 0.88, 95% CI 0.82–0.93, Z = 4.11, P<0.00001, I^2^ = 5%, Fig 3A), a trend for lower ICU morality (not statistically significant) was also observed in severely obese subjects (OR 0.82, 95% CI 0.66–1.00, Z = 1.92, P = 0.06, I^2^ = 35%, Fig 3A). Overweight and obese patients had lower long-term mortality (OR 0.66, 95% CI 0.57–0.77, Z = 5.38, P<0.00001, I^2^ = 0% and OR 0.63, 95% CI 0.46–0.86, Z = 2.88, P = 0.004, I^2^ = 56%, Fig 3B), only 1 of 23 studies reported long-term mortality in severely obese patients (lower than subjects with normal BMI) (Fig 3B). Obese and overweight patients had lower short-term mortality (OR 0.75, 95% CI 0.66–0.84, Z = 4.89, P<0.00001, I^2^ = 8% and OR 0.91, 95% CI 0.67–1.24, Z = 0.59, P = 0.55, I^2^ = 45%, Fig 3C). Obese and severely obese patients had lower hospital mortality (OR 0.80, 95% CI 0.73–0.89, Z = 4.22, P<0.0001, I^2^ = 30% and OR 0.71, 95% CI 0.53–0.94, Z = 2.40, P = 0.02, I^2^ = 74%, Fig 3D). In contrast, underweight subjects had higher mortality than normal BMI (ICU mortality in Fig 3A, long-term mortality in Fig 3B, short-term mortality in Fig 3C, hospital mortality in Fig 3D).

### Duration of mechanical ventilation

The mean duration of mechanical ventilation in the included studies ranged from 2.17 to 15.2 days in the obese subjects and 1.86 to 13.2 days in the non-obese subjects. In a sensitivity analysis that excluded the studies that did not report mean and standard deviation, the outcome did not significantly change. In comparison with non-obese subjects, the combined mean difference in duration of mechanical ventilation was longer by 0.48 days in obese patients (95% CI, 0.16–0.80, Z = 2.92, P = 0.003, I^2^ = 37%, S2A Fig). In comparison to the subjects with the normal BMI, severely obese patients had significantly longer duration on mechanical ventilation (MD 1.10, 95% CI 0.38–1.83, Z = 2.99, P = 0.003, I^2^ = 47%, S2B Fig). No significant differences were found between the normal BMI and underweight subjects (MD -0.26, 95% CI -0.89 to 0.38, Z = 0.79, P = 0.43, I^2^ = 11%, S2B Fig), overweight (MD 0.18, 95% CI -0.33 to 0.70, Z = 0.70, P = 0.48 I^2^ = 62%, S2B Fig) or obese patients (MD 0.23, 95% CI -0.03 to 0.48, Z = 1.75, P = 0.08, I^2^ = 0%, S2B Fig).

### ICU and hospital LOS

Non-obese patients had shorter ICU LOS than obese patients (MD 0.38, 95% CI 0.17–0.59, Z = 3.58, P = 0.0003, I^2^ = 70%, S2C Fig). A sensitivity analysis that excluded studies that did not report mean and standard deviation did not change these findings. In comparison to subjects with normal BMI, no significant differences in ICU LOS were found in underweight (MD -0.31, 95% CI -1.22 to 0.60, Z = 0.67, P = 0.50, I^2^ = 0%, S2D Fig), obese (MD 0.25, 95% CI -0.02 to 0.52, Z = 1.84, P = 0.07 I^2^ = 85%, S2D Fig), overweight (MD 0.09, 95% CI -0.04 to 0.21, Z = 1.37, P = 0.17, I^2^ = 48%, S2D Fig) and severely obese patients (MD 0.72, 95% CI -0.07 to 1.52, Z = 1.78, P = 0.08, I^2^ = 61%, S2D Fig). Hospital LOS did not differ between obese and non-obese patients (MD -0.06, 95% CI -0.52 0.63, Z = 0.19, P = 0.85, I^2^ = 80%, S2E Fig). A sensitivity analysis eliminating the studies that did not report mean and standard deviation did not change the findings. Hospital LOS did not differ among different BMI categories (S2F Fig).

## Discussion

Obesity is a risk factor of death in the general population [36]. The current meta-analysis showed that, in comparison to subjects with normal BMI, obese ICU patients receiving mechanical ventilation had lower measures of mortality rate (ICU, hospital, short-term as well as long-term). Several factors may have attributed to the “obesity paradox” in this population. First, adipose tissue is considered to be an ancestral immune organ [37], and could secrete leptin, adiponectin and many other biological response modifiers [38]. Leptin is a critical component of the host defense in the lungs [39]. Adiponectin produces anti-inflammatory effects through acting on inflammatory cells, NF-κB, and TNF-α, and could regulate inflammatory response, improve glucose tolerance and reduced vasopressor requirement in obese patients [40]. A recent study found that obese patients have lower levels of proinflammatory cytokines (IL-6, IL-8) and surfactant protein D, The lack of reduced mortality related to obesity might be due to low grade inflammatory response [41]. Second, both animal and human studies showed that adipocytes are infiltrated by activated macrophages under critical illnesses[41, 42]; these macrophages have important immune and scavenger functions, and produce an anti-inflammatory response. Third, obese patients have higher energy reservoir to counteract the influence by increased catabolic stress of disease [43]. Lastly, obese patients might have a lower threshold for ICU admission due to heightened perception of by doctors and nurses [20].

In the current study, underweight subjects had higher hospital mortality than those with normal BMI. Contributing factors may include insufficient energy stores to maintain organ function during times of critical illness and weak immune response to challenges due to poor nutritional status. A previous prospective study suggested low BMI as a potential marker for the underlying chronic diseases, such as cancer [44]. However, the increased risk of mortality reported in patients with a low BMI could also reflect illness-related weight loss or other serious illnesses before hospitalization [45].

Consistent with a previous study [46], duration on mechanical ventilation was longer in obese patients than in non-obese patients in the current study. Several reasons might have contributed to this finding. First, obese patients have decreased lung and chest wall compliance, and are more susceptible to atelectasis or increased alveolar tension [47]. Obese patients also tend to have ventilation flow imbalance, and lower functional residual and lung volume [48]. Second, obese patients consume more oxygen and produce more carbon dioxide production [49,50], and thus have increased respiratory work. Abdominal visceral fat accumulation could also increase abdominal pressure and thus increased respiratory work [51,52]. which were easy to cause respiratory muscle fatigue and difficult weaning. Third, clinicians often overestimate lung size for obese patients, and tend to use higher tidal volumes, thus placing patients on risk to develop ventilator-associated lung injury [21].

We also found significantly longer ICU LOS in obese patients than in non-obese patients. This could have been due to prolonged duration of mechanical ventilation in ICU, or higher rate of ICU complications, especially sepsis and pneumonia [14]. Also, nursing is particularly difficult in obese patients, and could lead to increased risks of skin laceration and other complications [53].

The current study has several limitations. First, assessment of BMI in the included studies could have been biased by resuscitation fluids given prior to ICU admission. Also, the height and weight of patients admitted into ICU typically estimated by physician rather than actually measured. Second, statistical heterogeneity in our analysis showed that the differences in outcomes might be explained by other characteristics not BMI, especially for length of stay (LOS) in hospital. Third, many confounding factors, including age, sex, and complications, could not be accurately extracted from the original studies. It must be emphasized that the results from the current study are based on ICU patients receiving mechanical ventilation. Similar results also occurred in obesity patients in ICU, Hogue [54] reported that obesity in ICU was associated with lower hospital mortality, but not associated with increased risk for ICU mortality. Extrapolation into other population must be cautious. Previous studies by Calle in the US [55] and Faeh in Switzerland [56] showed that obesity is associated with excess risk of mortality in adults, mainly due to complications, such as cardiovascular diseases and cancer. The existence of “obesity paradox” remains debated, and further studies are needed to determine whether adding BMI would decrease risk of mortality.

## Conclusion

In summary, compared to subjects with normal BMI, obese ICU subjects receiving mechanical ventilation had lower ICU and hospital mortality. There were also some evidence for lower short or long-term mortality. Obese patients had longer duration of mechanical ventilation and ICU LOS than non-obese patients.

## Supporting information

S1 Text MEDLINE search strategy. (PDF)

S1 Fig Funnel plots. (A) duration of mechanical ventilation.(B) ICU length of stay. (C) hospital length of stay.
(PDF)

S2 Fig Figures of duration of mechanical ventilation, ICU LOS and hospital LOS. (A) duration of mechanical ventilation in the obese vs non-obese patients.(B) duration of mechanical ventilation of different BMI classification.(C) ICU LOS of obese vs non-obese patients.(D) ICU LOS of different BMI classification.(E) hospital LOS of obese vs non-obese patients.(F) hospital LOS of different BMI classification.
(PDF)

S1 Table Quality assessment scale of included articles. (PDF)

S2 Table PRISMA checklist. (PDF)

## References

[1] Flegal KM, Kruszon-Moran D, Carroll MD, Fryar CD, Ogden CL.Trends in Obesity Among Adults in the United States, 2005 to 2014. Jama. 2016;315(21):2284 doi: 10.1001/jama.2016.6458 . 27272580 10.1001/jama.2016.6458 PMC11197437

[2] Arabi YM, Dara SI, Tamim HM, Rishu AH, Bouchama A, Khedr MK, et al Clinical characteristics, sepsis interventions and outcomes in the obese patients with septic shock: an international multicenter cohort study. Crit Care. 2013;17(2):R72 doi: 10.1186/cc12680 . 23594407 10.1186/cc12680 PMC3672731

[3] Obesity: preventing and managing the global epidemic. Report of a WHO consultation. World Health Organ Tech Rep Ser. 2000;894:1–253. . 11234459

[4] Wang S, Ma W, Wang S, Yi X, Jia H, Xue F. Obesity and Its Relationship with Hypertension among Adults 50 Years and Older in Jinan, China. Plos One. 2014;9(12):e114424 doi: 10.1371/journal.pone.0114424 . 25517735 10.1371/journal.pone.0114424 PMC4269412

[5] Gortmaker SL, Swinburn BA, Levy D, Carter R, Mabry PL, Finegood DT, et al Changing the future of obesity: science, policy, and action. The Lancet. 2011;378(9793):838–847. doi: 10.1016/S0140-6736(11)60815-5 . 21872752 10.1016/S0140-6736(11)60815-5 PMC3417037

[6] Hossain P, Kawar B, El NM. Obesity and diabetes in the developing world-a growing challenge. N Engl J Med. 2007;356(3):213–5. doi: 10.1056/NEJMp068177 . 17229948 10.1056/NEJMp068177

[7] Lewandowski K, Lewandowski M. Intensive care in the obese. Best Practice & Research Clinical Anaesthesiology. 2011;25(1):95–108. https://doi.org/10.1016/j.bpa.2010.12.003 . 21516917 10.1016/j.bpa.2010.12.003

[8] Goulenok C, Monchi M, Chiche JD, Mira JP, Dhainaut JF, Cariou A. Influence of overweight on ICU mortality: a prospective study. CHEST. 2004;125(4): 1441–1445. . 15078757 10.1378/chest.125.4.1441

[9] Brown CVR, Neville AL, Rhee P, Salim A, Velmahos GC, Demetriades D. The Impact of Obesity on the Outcomes of 1,153 Critically Injured Blunt Trauma Patients. The Journal of Trauma: Injury, Infection, and Critical Care.2005:1048–1051.. 16385276 10.1097/01.ta.0000189047.65630.c5

[10] El-Solh A, Sikka P, Bozkanat E, Jaafar W, Davies J. Morbid obesity in the medical ICU. Chest.2001;120(6):1989–1997. https://doi.org/10.1378/chest.120.6.1989 . 11742933 10.1378/chest.120.6.1989

[11] Katzmarzyk PT, Reeder BA, Elliott S, Joffres MR, Pahwa P, Raine KD, et al Body mass index and risk of cardiovascular disease, cancer and all-cause mortality. Can J Public Health. 2012;103(2):147–151.. 22530540 10.1007/BF03404221 PMC6974265

[12] Trivedi V, Jean RE, Genese F, Fuhrmann KA, Saini AK, Mangulabnan VD, et al Impact of Obesity on Outcomes in a Multiethnic Cohort of Medical Intensive Care Unit Patients. J Intensive Care Med. 2016;33(2):97–103. doi: 10.1177/0885066616646099 . 27139008 10.1177/0885066616646099

[13] O'Brien JM, Philips GS, Ali NA, Aberegg SK, Marsh CB, Lemeshow S. The association between body mass index, processes of care, and outcomes from mechanical ventilation. Crit Care Med. 2012;40(5):1456–1463. doi: 10.1097/CCM.0b013e31823e9a80 . 22430246 10.1097/CCM.0b013e31823e9a80

[14] Dickerson RN. The obesity paradox in the ICU: real or not? Critical care (London, England). 2013;17(3):154 doi: 10.1186/cc12715 . 23758984 10.1186/cc12715 PMC3706970

[15] Clinical Guidelines on the Identification, Evaluation, and Treatment of Overweight and Obesity in Adults-The Evidence Report. Am J Clin Nutr. 1998 10;68(4):899–917. doi: 10.1093/ajcn/68.4.899 . 9771869 10.1093/ajcn/68.4.899

[16] DerSimonian R, Laird N. Meta-analysis in clinical trials. Control Clin Trials. 1986;7:177–188. . 3802833 10.1016/0197-2456(86)90046-2

[17] Higgins JPT, Green S. Cochrane handbook for Systematic Reviews of Interventions Version 5.1.0. The Cochrane Collaboration,2011. Available from http://handbook.cochrane.org.

[18] Tafelski S, Yi H, Ismaeel F, Krannich A, Spies C, Nachtigall I. Obesity in critically ill patients is associated with increased need of mechanical ventilation but not with mortality. J Infect Public Health. 2016;9(5):577–585. doi: 10.1016/j.jiph.2015.12.003 . 26754202 10.1016/j.jiph.2015.12.003

[19] Wardell S, Wall A, Bryce R, Gjevre JA, Laframboise K, Reid JK. The association between obesity and outcomes in critically ill patients. Can Respir J.2015;22(1):23–30. doi: 10.1155/2015/938930 . 25379653 10.1155/2015/938930 PMC4324521

[20] Martino JL, Stapleton RD, Wang M, Day AG, Cahill NE, Dixon AE, et al Extr- eme obesity and outcomes in critically ill patients. Chest. 2011;140(5):1198–206. doi: 10.1378/chest.10-3023 . 21816911 10.1378/chest.10-3023 PMC3205847

[21] Anzueto A, Frutos-Vivar F, Esteban A, Bensalami N, Marks D, Raymondos K, et al Influence of body mass index on outcome of the mechanically ventilated patients. Thorax. 2011;66(1):66–73. doi: 10.1136/thx.2010.145086 . 20980246 10.1136/thx.2010.145086

[22] Diaz E, Rodriguez A, Martin-Loeches I, Lorente L, Del MMM, Pozo JC, et al Impact of obesity in patients infected with 2009 influenza A(H1N1). Chest. 2011;139(2):382–386. doi: 10.1378/chest.10-1160 . 20688928 10.1378/chest.10-1160

[23] Alberda C, Gramlich L, Jones N, Jeejeebhoy K, Day AG, Dhaliwal R, et al The relationship between nutritional intake and clinical outcomes in critically ill patients: results of an international multicenter observational study. Intensive Care Med. 2009;35(10):1728–1737. doi: 10.1007/s00134-009-1567-4 . 19572118 10.1007/s00134-009-1567-4

[24] Frat J, Gissot V, Ragot S, Desachy A, Runge I, Lebert C, et al Impact of obesity in mechanically ventilated patients: a prospective study. Intensive Care Med. 2008;34(11):1991–8. doi: 10.1007/s00134-008-1245-y . 18670754 10.1007/s00134-008-1245-y

[25] Morris AE, Stapleton RD, Rubenfeld GD, Hudson LD, Caldwell E, Steinberg KP. The association between body mass index and clinical outcomes in acute lung injury. Chest. 2007;131(2):342–348. doi: 10.1378/chest.06-1709 . 17296631 10.1378/chest.06-1709

[26] Peake SL, Moran JL, Ghelani DR, Lloyd AJ, Walker MJ. The effect of obesity on 12-month survival following admission to intensive care: a prospective study. Crit Care Med. 2006;34(12):2929–2939. doi: 10.1097/01.CCM.0000248726.75699.B1 . 17075374 10.1097/01.CCM.0000248726.75699.B1

[27] Ray DE, Matchett SC, Baker K, Wasser T, Young MJ. The Effect of Body Mass In-dex on Patient Outcomes in a Medical ICU. Chest. 2005;127(6):2125–2131. doi: 10.1378/chest.127.6.2125 . 15947330 10.1378/chest.127.6.2125

[28] Abhyankar S, Leishear K, Callaghan FM, Demner-Fushman D, McDonald CJ. Lower short- and long-term mortality associated with overweight and obesity in a large cohort study of adult intensive care unit patients. Crit Care. 2012;16(6):R235 doi: 10.1186/cc11903 . 23249446 10.1186/cc11903 PMC3672624

[29] Pickkers P, de Keizer N, Dusseljee J, Weerheijm D, van der Hoeven JG, Peek N. Body mass index is associated with hospital mortality in critically ill patients: an observational cohort study. Crit Care Med. 2013;41(8):1878–1883. doi: 10.1097/CCM.0b013e31828a2aa1 . 23685638 10.1097/CCM.0b013e31828a2aa1

[30] Lee CK, Tefera E, Colice G.The effect of obesity on outcomes in mechanically ventilated patients in a medical intensive care unit. Respiration. 2014;87(3):219–226. doi: 10.1159/000357317 . 24457313 10.1159/000357317

[31] Moock M, Mataloun SE, Pandolfi M, Coelho J, Novo N, Compri PC. Impact of obesity on critical care treatment in adult patients. Rev Bras Ter Intensiva. 2010;22(2):133–137. . 25303754

[32] Duane TM, Dechert T, Aboutanos MB, Malhotra AK, Ivatury RR. Obesity and out-comes after blunt trauma. J Trauma. 2006;61(5):1218–1221. doi: 10.1097/01.ta.0000241022.43088.e1 . 17099532 10.1097/01.ta.0000241022.43088.e1

[33] O'Brien JM Jr, Phillips GS, Ali NA, Lucarelli M, Marsh CB. Body mass index is independently associated with hospital mortality in mechanically ventilated adults with acute lung injury. Critical Care Medicine. 2006; 34(3): 738–744. doi: 10.1097/01.CCM.0000202207.87891.FC . 16521268 10.1097/01.CCM.0000202207.87891.FC PMC1868702

[34] Lewis O, Ngwa J, Kibreab A, Phillpotts M, Thomas A, Mehari A. Body Mass Index and Intensive Care Unit Outcomes in African American Patients. Ethn Dis. 2017;27(2):161–168. doi: 10.18865/ed.27.2.161 . 28439187 10.18865/ed.27.2.161 PMC5398175

[35] Dennis DM, Bharat C, Paterson T. Prevalence of obesity and the effect on length of mechanical ventilation and length of stay in intensive care patients: A single site observational study. Australian Critical Care.2016;30(3):145–150. doi: 10.1016/j.aucc.2016.07.003 . 27522470 10.1016/j.aucc.2016.07.003

[36] Adams KF, Schatzkin A, Harris TB, Kipnis V, Mouw T, Ballard-Barbash R, et al Overweight, obesity, and mortality in a large prospective cohort of persons 50 to 71 years old. N Engl J Med. 2006;355(8):763–778. doi: 10.1056/NEJMoa055643 . 16926275 10.1056/NEJMoa055643

[37] Satoh M, Iwabuchi K. Communication between natural killer T cells and adipocytes in obesity. Adipocyte. 2016 9 29;5(4):389–393. doi: 10.1080/21623945.2016.1241913 . 27994954 10.1080/21623945.2016.1241913 PMC5160409

[38] Waki H, Tontonoz P. Endocrine functions of adipose tissue. Annual Review of Pathology. 2007;2 (2):31–56. doi: 10.1146/annurev.pathol.2.010506.091859 . 18039092 10.1146/annurev.pathol.2.010506.091859

[39] Mancuso P, Gottschalk A, Phare SM, Peters-Golden M, Lukacs NW, Huffnagle GB. Leptin-deficient mice exhibit impaired host defense in gram-negative pneumonia. The Journal of Immunology.2002;168(8):4018–4024. https://doi.org/10.4049/jimmunol.168.8.4018 . 11937559 10.4049/jimmunol.168.8.4018

[40] Robinson K, Prins J. and Venkatesh B., Clinical review: adiponectin biology and its role in inflammation and critical illness. Crit Care. 2011;15(2): 221–230. doi: 10.1186/cc10021 . 21586104 10.1186/cc10021 PMC3219307

[41] Stapleton RD, Dixon AE, Parsons PE, Ware LB, Suratt BT, NHLBI. The association between BMI and plasma cytokine levels in patients with acute lung injury. Chest. 2010; 138:568–577. doi: 10.1378/chest.10-0014 . 20435656 10.1378/chest.10-0014 PMC2940070

[42] Weisberg SP, McCann D, Desai M, Rosenbaum M, Leibel RL, Ferrante AW Jr. Obesity is associated with macrophage accumulation in adipose tissue.J Clin Invest. 2003;112:1796–1808. doi: 10.1172/JCI19246 . 14679176 10.1172/JCI19246 PMC296995

[43] Zhi G, Xin W, Ying W, Guohong X, Shuying L. “Obesity Paradox” in Acute Respiratory DistressSyndrome: Asystematic Review and Meta-Analysis. PLOS ONE. 2016;11(9):e163677 doi: 10.1371/journal.pone.0163677 . 27684705 10.1371/journal.pone.0163677 PMC5042414

[44] Garrouste-Orgeas M, Troché G, Azoulay E, Caubel A, de Lassence A, Cheval C, et al Body mass index. An additional prognostic factor in ICU patients. Intensive Care Med. 2004;30(3):437–443. doi: 10.1007/s00134-003-2095-2 . 14767583 10.1007/s00134-003-2095-2

[45] Wannamethee SG, Shaper AG, Walker M. Weight change, body weight and mortality:the impact of smoking and ill health. Int J Epidemiol. 2001;30:777–786. . 11511602 10.1093/ije/30.4.777

[46] Akinnusi ME, Pineda LA, El Solh AA. Effect of obesity on intensive care morbidity and mortality: A meta-analysis. Crit Care Med. 2008;36(1):151–158. doi: 10.1097/01.CCM.0000297885.60037.6E . 18007266 10.1097/01.CCM.0000297885.60037.6E

[47] Pelosi P, Croci M, Ravagnan I, Vicardi P, Gattinoni L. Total respiratory system, lung, and chest wall mechanics in sedated-paralyzed postoperative morbidly obese patients.Chest.1996;109:144–151.. 8549177 10.1378/chest.109.1.144

[48] Salome CM, King GG, Berend N. Physiology of obesity and effects on lung function. J Appl Physiol. 2010; 108:206–211. doi: 10.1152/japplphysiol.00694.2009 . 19875713 10.1152/japplphysiol.00694.2009

[49] Kress JP, Pohlman AS, Alverdy J, Hall JB. The impact of morbid obesity on oxygen cost of breathing (VO(2RESP)) at rest. Am J Respir Crit Care Med. 1999;160:883–886. doi: 10.1164/ajrccm.160.3.9902058 . 10471613 10.1164/ajrccm.160.3.9902058

[50] De Jong A, Chanques G, Jaber S.Mechanical ventilation in obese ICU patients: from intubation to extubation. Crit Care. 2017;21(1):63 doi: 10.1186/s13054-017-1641-1 . 28320439 10.1186/s13054-017-1641-1 PMC5359820

[51] Pepin J, Borel JC, Janssens JP. Obesity hypoventilation syndrome:an underdiagnosed and undertreated condition. Am J Respir Crit Care Med.2012;186:1205–1207. doi: 10.1164/rccm.201210-1922ED . 23250497 10.1164/rccm.201210-1922ED

[52] Chlif M, Keochkerian D, Choquet D, Vaidie A, Ahmaidi S. Effects of obesity on breathing pattern, ventilatory neural drive and mechanics. Respir Physiol Neurobiol. 2009;168:198–202. doi: 10.1016/j.resp.2009.06.012 . 19559105 10.1016/j.resp.2009.06.012

[53] Winkelman C, Maloney B, Kloos J. The impact of obesity on critical care resource use and outcomes. Crit Care Nurs Clin North Am. 2009;21(3):403–422. doi: 10.1016/j.ccell.2009.07.002 . 19840718 10.1016/j.ccell.2009.07.002

[54] Hogue CJ, Stearns JD, Colantuoni E, Robinson KA, Stierer T, Mitter N, et al The impact of obesity on outcomes after critical illness: a meta-analysis. Intensive Care Med.2009 2009;35(7):1152–1170. doi: 10.1007/s00134-009-1424-5 19189078 10.1007/s00134-009-1424-5

[55] Calle EE, Thun MJ, Petrelli JM, Rodriguez C, Heath CW Jr. Body-mass index and mortality in a prospective cohort of U.S. adults. N Engl J Med. 1999;341(15):1097–1105. doi: 10.1056/NEJM199910073411501 . 10511607 10.1056/NEJM199910073411501

[56] Faeh D, Braun J, Tarnutzer S, Bopp M. Obesity but not overweight is associated with increased mortality risk. Eur J Epidemiol. 2011;26(8):647–655. doi: 10.1007/s10654-011-9593-2 . 21681546 10.1007/s10654-011-9593-2

