# Supplementary material for: Is body mass index associated with outcomes of mechanically ventilated adult patients in intensive critical units? A systematic review and meta-analysis
Source: PLoS One. 2018 Jun 8;13(6):e0198669. doi: 10.1371/journal.pone.0198669 (PMC5993298; doi:10.1371/journal.pone.0198669)
Supplement: S1 Text — (PDF) [file pone.0198669.s001.pdf]

Database search strategies

1. MEDLINE

2. EMBASE

3. Cochrane Central Register of Controlled Trials (CENTRAL)

MEDLINE

1. **"Respiration, Artificial"[Mesh] OR "Ventilators, Mechanical"[Mesh]**
2. **ventilator[Title/Abstract] OR mechanically ventilated[Title/Abstract]) OR mechanical ventilation[Title/Abstract]**
3. **1 or 2**
4. **"Body Weight"[Mesh] OR "Overweight"[Mesh]) OR "Obesity"[Mesh]**
5. **body weight[Title/Abstract] OR BMI[Title/Abstract]) OR body mass index[Title/Abstract]) OR overweight[Title/Abstract]) OR underweight[Title/Abstract]) OR obesity[Title/Abstract]) OR Obese[Title/Abstract]**
6. **4 or 5**
7. **3 and 6**
