# Supplementary material for: Is body mass index associated with outcomes of mechanically ventilated adult patients in intensive critical units? A systematic review and meta-analysis
Source: PLoS One. 2018 Jun 8;13(6):e0198669. doi: 10.1371/journal.pone.0198669 (PMC5993298; doi:10.1371/journal.pone.0198669)
Supplement: S1 Fig — (A) duration of mechanical ventilation.(B) ICU length of stay. (C) hospital length of stay. (PDF) [file pone.0198669.s002.pdf]

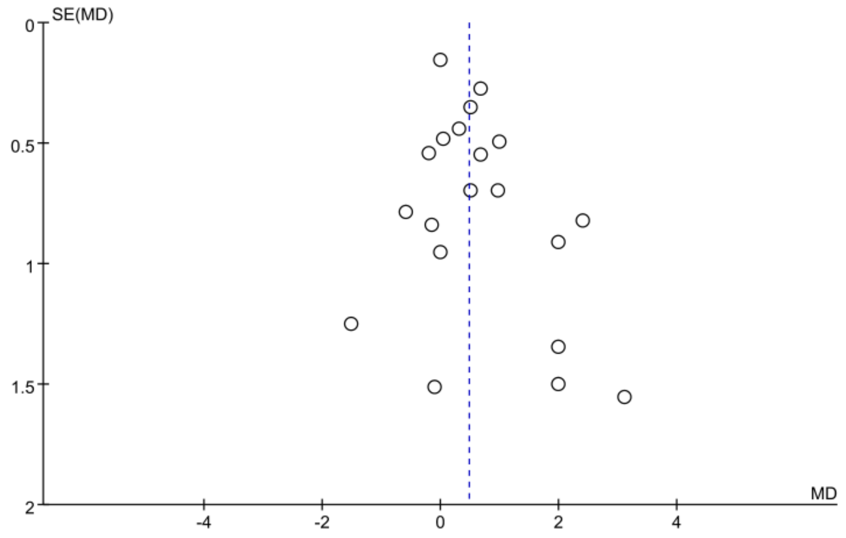

A. Funnel plot assessing publication bias of duration of mechanical ventilation

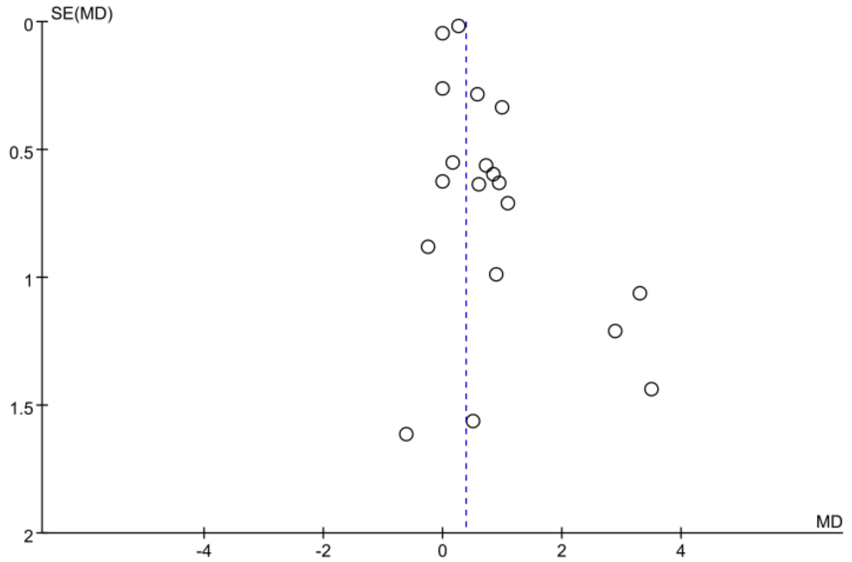

B. Funnel plot assessing publication bias of ICU length of stay

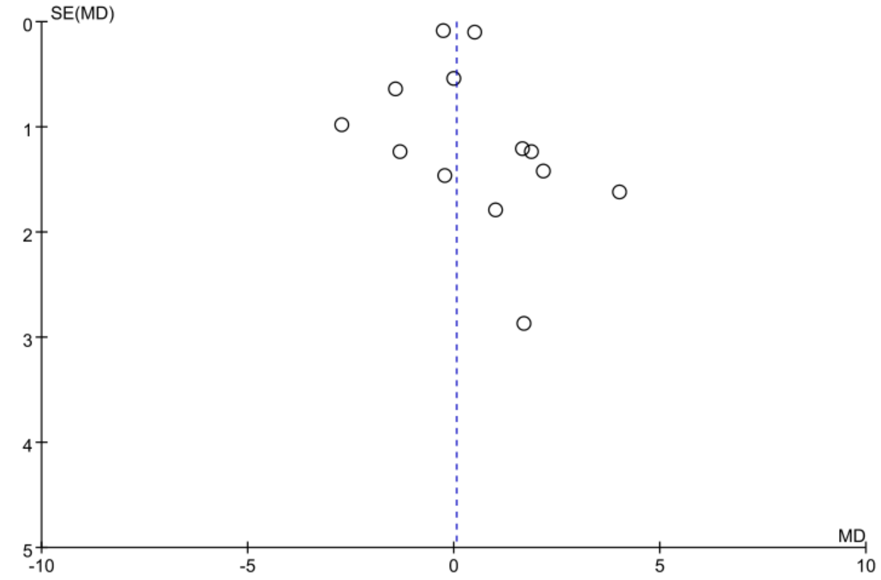

C. Funnel plot assessing publication bias of hospital length of stay
