# Supplementary material for: Is body mass index associated with outcomes of mechanically ventilated adult patients in intensive critical units? A systematic review and meta-analysis
Source: PLoS One. 2018 Jun 8;13(6):e0198669. doi: 10.1371/journal.pone.0198669 (PMC5993298; doi:10.1371/journal.pone.0198669)
Supplement: S2 Fig — (A) duration of mechanical ventilation in the obese vs non-obese patients.(B) duration of mechanical ventilation of different BMI classification.(C) ICU LOS of obese vs non-obese patients.(D) ICU LOS of different BMI classification.(E) hospital LOS of obese vs non-obese patients.(F) hospital LOS of different BMI classification. (PDF) [file pone.0198669.s003.pdf]

| Study or Subgroup | obese Mean | obese SD | obese Total | non-obese Mean | non-obese SD | non-obese Total | Weight | Mean Difference IV, Random, 95% CI |
|---|---|---|---|---|---|---|---|---|
| **1.1.1 Duration of Mechanical Ventilation** | | | | | | | | |
| Alberda C, 2009 | 9.51 | 12.75 | 728 | 8.83 | 11.68 | 1755 | 5.9% | 0.68 [-0.40, 1.76] |
| Anzueto A, 2011 | 4 | 4.44 | 1008 | 4 | 4.44 | 3776 | 15.3% | 0.00 [-0.31, 0.31] |
| Brown CV, 2005 | 8 | 13 | 230 | 6 | 9 | 870 | 2.7% | 2.00 [0.22, 3.78] |
| Diane M.D,2016 | 2.81 | 4.54 | 268 | 2.3 | 4.64 | 449 | 9.7% | 0.51 [-0.18, 1.20] |
| Diaz E, 2011 | 15.2 | 8.9 | 96 | 13.2 | 11.7 | 139 | 1.4% | 2.00 [-0.64, 4.64] |
| Duane TM, 2006 | 3.7 | 4 | 10 | 3.8 | 5.9 | 51 | 1.1% | -0.10 [-3.06, 2.86] |
| El-Solh A, 2001 | 7.7 | 10.6 | 71 | 4.6 | 7.1 | 61 | 1.0% | 3.10 [0.06, 6.14] |
| Frat JP, 2008 | 9 | 5.93 | 82 | 10.5 | 11.85 | 124 | 1.6% | -1.50 [-3.95, 0.95] |
| Goulenok C, 2004 | 4 | 5.19 | 142 | 3 | 4.44 | 353 | 6.8% | 1.00 [0.03, 1.97] |
| Lee CK, 2014 | 6.1 | 8.1 | 198 | 5.6 | 6.9 | 306 | 4.2% | 0.50 [-0.87, 1.87] |
| Martino JL, 2011 | 7.97 | 11.79 | 2296 | 7.3 | 9.96 | 6094 | 11.8% | 0.67 [0.13, 1.21] |
| Moock M, 2010 | 7 | 8.15 | 43 | 5 | 7.04 | 70 | 1.1% | 2.00 [-0.94, 4.94] |
| Morris AE, 2007 | 8.87 | 9.58 | 237 | 9.45 | 11.08 | 538 | 3.5% | -0.58 [-2.12, 0.96] |
| O'Brien JM Jr, 2012 | 5 | 6.3 | 238 | 5.19 | 6.57 | 342 | 6.0% | -0.19 [-1.25, 0.87] |
| O'Dene Lewis,2017 | 8.63 | 7.78 | 211 | 7.67 | 8.18 | 333 | 4.2% | 0.96 [-0.41, 2.33] |
| Peake SL, 2007 | 2.17 | 3.85 | 106 | 1.86 | 3.44 | 216 | 7.8% | 0.31 [-0.55, 1.17] |
| Ray DE, 2005 | 5.17 | 6.23 | 239 | 5.12 | 5.9 | 490 | 7.0% | 0.05 [-0.90, 1.00] |
| S. Tafelski, 2016 | 3 | 6.67 | 85 | 3 | 9.62 | 240 | 2.5% | 0.00 [-1.87, 1.87] |
| Trivedi,2017 | 4 | 8.1 | 205 | 4.15 | 13.16 | 453 | 3.1% | -0.15 [-1.79, 1.49] |
| Wardell S, 2015 | 6.5 | 8.8 | 146 | 4.1 | 5.5 | 202 | 3.2% | 2.40 [0.78, 4.02] |
| **Subtotal (95% CI)** | | | **6639** | | | **16862** | **100.0%** | **0.48 [0.16, 0.80]** |
| Heterogeneity: Tau² = 0.15; Chi² = 30.17, df = 19 (P = 0.05); I² = 37% | | | | | | | | |
| Test for overall effect: Z = 2.92 (P = 0.003) | | | | | | | | |

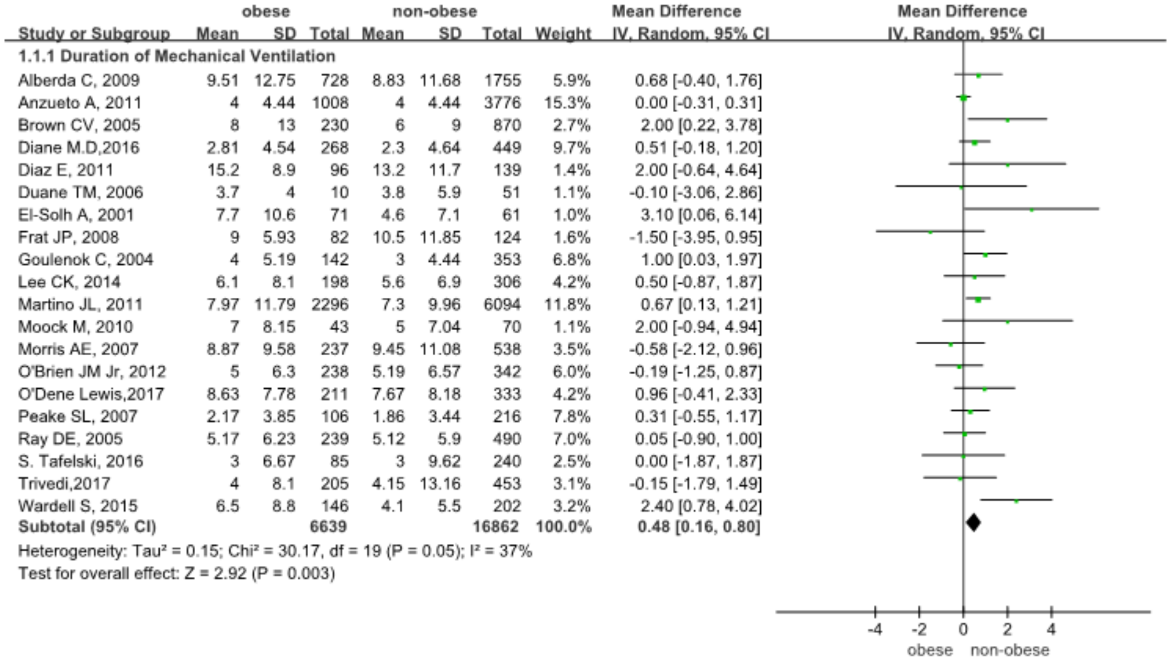


**A. duration of mechanical ventilation in the obese vs non-obese patients**

| Study or Subgroup | Experimental Mean | Experimental SD | Experimental Total | Control Mean | Control SD | Control Total | Weight | Mean Difference IV, Random, 95% CI |
|---|---|---|---|---|---|---|---|---|
| **2.1.1 obese vs normal** | | | | | | | | |
| Morris AE, 2007 | 8.3 | 8.4 | 183 | 8.7 | 10.7 | 301 | 2.2% | -0.40 [-2.12, 1.32] |
| O'Dene Lewis,2017 | 7.58 | 7.25 | 204 | 7.75 | 9.05 | 206 | 2.6% | -0.17 [-1.76, 1.42] |
| Anzueto A, 2011 | 4 | 4.44 | 792 | 4 | 4.44 | 1995 | 48.9% | 0.00 [-0.37, 0.37] |
| O'Brien JM Jr, 2012 | 5 | 6.3 | 238 | 5 | 6.7 | 213 | 4.5% | 0.00 [-1.20, 1.20] |
| Ray DE, 2005 | 5.2 | 6.6 | 168 | 5 | 5.9 | 266 | 4.4% | 0.20 [-1.02, 1.42] |
| Peake SL, 2007 | 1.76 | 3.18 | 64 | 1.5 | 3.89 | 97 | 5.4% | 0.26 [-0.84, 1.36] |
| Alberda C, 2009 | 9.11 | 12.62 | 557 | 8.6 | 10.89 | 937 | 4.1% | 0.51 [-0.75, 1.77] |
| Martino JL, 2011 | 7.9 | 11.33 | 1772 | 7.3 | 10.37 | 3490 | 16.5% | 0.60 [-0.03, 1.23] |
| Diane M.D,2016 | 2.65 | 4.43 | 216 | 1.79 | 3.38 | 200 | 11.5% | 0.86 [0.11, 1.61] |
| **Subtotal (95% CI)** | | | **4194** | | | **7705** | **100.0%** | **0.23 [-0.03, 0.48]** |
| Heterogeneity: Tau² = 0.00; Chi² = 6.63, df = 8 (P = 0.58); I² = 0% | | | | | | | | |
| Test for overall effect: Z = 1.75 (P = 0.08) | | | | | | | | |
| **2.1.2 overweight vs normal** | | | | | | | | |
| Trivedi,2017 | 2.5 | 6.1 | 194 | 5.4 | 16.5 | 259 | 4.7% | -2.90 [-5.09, -0.71] |
| O'Dene Lewis,2017 | 7.56 | 6.57 | 127 | 7.75 | 9.05 | 206 | 7.2% | -0.19 [-1.87, 1.49] |
| Martino JL, 2011 | 7.3 | 9.41 | 2604 | 7.3 | 10.37 | 3490 | 23.5% | 0.00 [-0.50, 0.50] |
| Anzueto A, 2011 | 4 | 4.44 | 1781 | 4 | 4.44 | 1995 | 27.6% | 0.00 [-0.28, 0.28] |
| Alberda C, 2009 | 9.1 | 12.52 | 818 | 8.6 | 10.89 | 937 | 12.6% | 0.50 [-0.61, 1.61] |
| Diane M.D,2016 | 2.71 | 4.65 | 249 | 1.79 | 3.38 | 200 | 18.5% | 0.92 [0.18, 1.66] |
| Morris AE, 2007 | 10.4 | 11.5 | 237 | 8.7 | 10.7 | 301 | 5.9% | 1.70 [-0.20, 3.60] |
| **Subtotal (95% CI)** | | | **6010** | | | **7388** | **100.0%** | **0.18 [-0.33, 0.70]** |
| Heterogeneity: Tau² = 0.23; Chi² = 15.89, df = 6 (P = 0.01); I² = 62% | | | | | | | | |
| Test for overall effect: Z = 0.70 (P = 0.48) | | | | | | | | |
| **2.1.3 severe obesity vs normal** | | | | | | | | |
| Anzueto A, 2011 | 4 | 4.44 | 216 | 4 | 4.44 | 1995 | 22.8% | 0.00 [-0.62, 0.62] |
| Ray DE, 2005 | 5.1 | 5.3 | 71 | 5 | 5.9 | 266 | 13.3% | 0.10 [-1.32, 1.52] |
| Martino JL, 2011 | 8.2 | 13.25 | 524 | 7.3 | 10.37 | 3490 | 15.8% | 0.90 [-0.29, 2.09] |
| Peake SL, 2007 | 2.79 | 4.66 | 42 | 1.5 | 3.89 | 97 | 11.7% | 1.29 [-0.32, 2.90] |
| Diane M.D,2016 | 3.5 | 4.99 | 52 | 1.79 | 3.38 | 200 | 13.2% | 1.71 [0.28, 3.14] |
| Morris AE, 2007 | 10.8 | 12.7 | 54 | 8.7 | 10.7 | 301 | 3.5% | 2.10 [-1.50, 5.70] |
| Alberda C, 2009 | 10.8 | 13.11 | 171 | 8.6 | 10.89 | 937 | 8.4% | 2.20 [0.11, 4.29] |
| O'Dene Lewis,2017 | 10.36 | 8.7 | 64 | 7.75 | 9.05 | 206 | 6.6% | 2.61 [0.15, 5.07] |
| El-Solh A, 2001 | 7.7 | 10.6 | 71 | 4.7 | 7.1 | 61 | 4.7% | 3.00 [-0.04, 6.04] |
| **Subtotal (95% CI)** | | | **1265** | | | **7553** | **100.0%** | **1.10 [0.38, 1.83]** |
| Heterogeneity: Tau² = 0.50; Chi² = 15.10, df = 8 (P = 0.06); I² = 47% | | | | | | | | |
| Test for overall effect: Z = 2.99 (P = 0.003) | | | | | | | | |
| **2.1.4 underweight vs normal** | | | | | | | | |
| O'Dene Lewis,2017 | 5.67 | 5.87 | 61 | 7.75 | 9.05 | 206 | 10.2% | -2.08 [-4.00, -0.16] |
| Trivedi,2017 | 3.4 | 8.4 | 41 | 5.4 | 16.5 | 259 | 3.7% | -2.00 [-5.26, 1.26] |
| Diane M.D,2016 | 1.7 | 3.08 | 18 | 1.79 | 3.38 | 200 | 16.0% | -0.09 [-1.59, 1.41] |
| Anzueto A, 2011 | 4 | 4.44 | 184 | 4 | 4.44 | 1995 | 53.8% | 0.00 [-0.67, 0.67] |
| Alberda C, 2009 | 8.8 | 13.56 | 289 | 8.6 | 10.89 | 937 | 12.6% | 0.20 [-1.51, 1.91] |
| Morris AE, 2007 | 9.2 | 10.9 | 50 | 8.7 | 10.7 | 301 | 3.7% | 0.50 [-2.75, 3.75] |
| **Subtotal (95% CI)** | | | **643** | | | **3898** | **100.0%** | **-0.26 [-0.89, 0.38]** |
| Heterogeneity: Tau² = 0.08; Chi² = 5.59, df = 5 (P = 0.35); I² = 11% | | | | | | | | |
| Test for overall effect: Z = 0.79 (P = 0.43) | | | | | | | | |

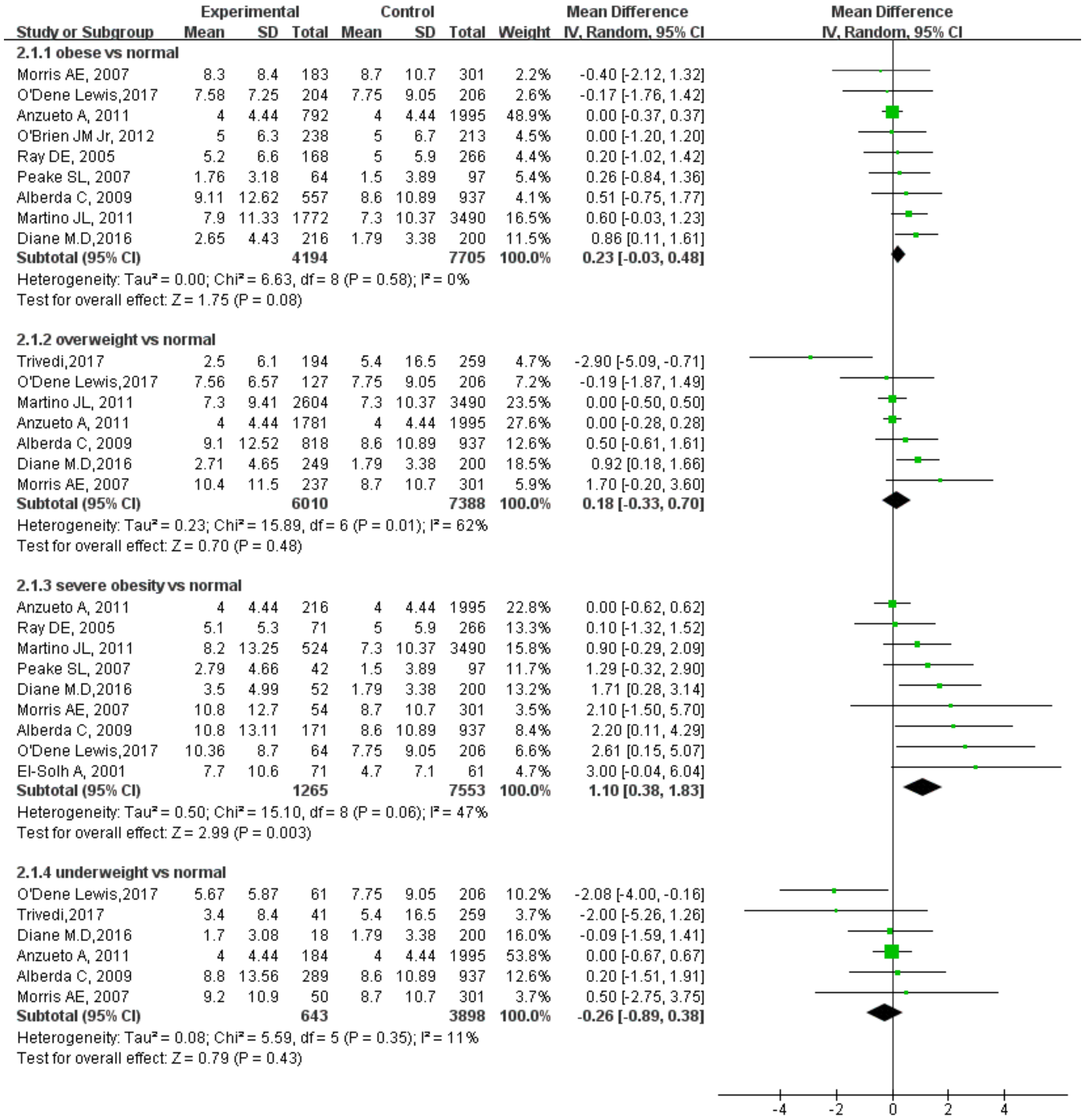


**B. duration of mechanical ventilation of different BMI classification**

| Study or Subgroup | obese Mean | obese SD | obese Total | non-obese Mean | non-obese SD | non-obese Total | Weight | Mean Difference IV, Random, 95% CI |
|---|---|---|---|---|---|---|---|---|
| Alberda C, 2009 | 12.2 | 12.71 | 728 | 12.03 | 11.88 | 1755 | 3.3% | 0.17 [-0.91, 1.25] |
| Anzueto A, 2011 | 8 | 7.41 | 1008 | 8 | 7.81 | 3776 | 9.9% | 0.00 [-0.52, 0.52] |
| Diane M.D,2016 | 6.39 | 7.78 | 268 | 5.55 | 7.78 | 449 | 2.8% | 0.84 [-0.34, 2.02] |
| Diaz E, 2011 | 13.7 | 11.7 | 150 | 10.8 | 12.1 | 265 | 0.8% | 2.90 [0.53, 5.27] |
| Duane TM, 2006 | 4.4 | 4.2 | 10 | 5 | 6.5 | 51 | 0.4% | -0.60 [-3.76, 2.56] |
| El-Solh A, 2001 | 9.3 | 13.5 | 117 | 5.8 | 8.2 | 132 | 0.5% | 3.50 [0.68, 6.32] |
| Frat JP, 2008 | 13.5 | 7.41 | 82 | 13 | 14.81 | 124 | 0.5% | 0.50 [-2.56, 3.56] |
| Goulenok C, 2004 | 4 | 4.44 | 215 | 3 | 3.7 | 598 | 7.1% | 1.00 [0.34, 1.66] |
| Lee CK, 2014 | 6.5 | 7.3 | 198 | 5.9 | 6.5 | 306 | 2.5% | 0.60 [-0.65, 1.85] |
| Martino JL, 2011 | 11.41 | 11.96 | 2296 | 10.83 | 11.03 | 6094 | 9.0% | 0.58 [0.02, 1.14] |
| Moock M, 2010 | 3.6 | 6.37 | 73 | 2.7 | 7.85 | 146 | 1.1% | 0.90 [-1.04, 2.84] |
| Morris AE, 2007 | 11.75 | 10.97 | 237 | 11.99 | 12.04 | 538 | 1.4% | -0.24 [-1.97, 1.49] |
| O'Brien, JM, 2006 | 12.53 | 12.58 | 457 | 11.43 | 12.28 | 943 | 2.1% | 1.10 [-0.29, 2.49] |
| O'Dene Lewis,2017 | 6.42 | 7.5 | 211 | 5.48 | 6.66 | 333 | 2.6% | 0.94 [-0.30, 2.18] |
| Peter Pickkers,2013 | 1.5 | 2.52 | 21991 | 1.24 | 2.24 | 127024 | 25.1% | 0.26 [0.22, 0.30] |
| S. Tafelski, 2016 | 6 | 5.93 | 130 | 6 | 7.41 | 451 | 2.6% | 0.00 [-1.23, 1.23] |
| Swapna Abhyankar,2012 | 2.3 | 2.74 | 5287 | 2.3 | 2.63 | 10739 | 24.2% | 0.00 [-0.09, 0.09] |
| Trivedi,2017 | 6.1 | 6.6 | 205 | 5.36 | 6.98 | 453 | 3.1% | 0.74 [-0.37, 1.85] |
| Wardell S, 2015 | 9.3 | 11.7 | 146 | 6 | 6.2 | 202 | 1.0% | 3.30 [1.22, 5.38] |
| **Total (95% CI)** | | | **33809** | | | **154379** | **100.0%** | **0.38 [0.17, 0.59]** |

Heterogeneity: Tau² = 0.05; Chi² = 60.30, df = 18 (P < 0.00001); I² = 70%

Test for overall effect: Z = 3.58 (P = 0.0003)

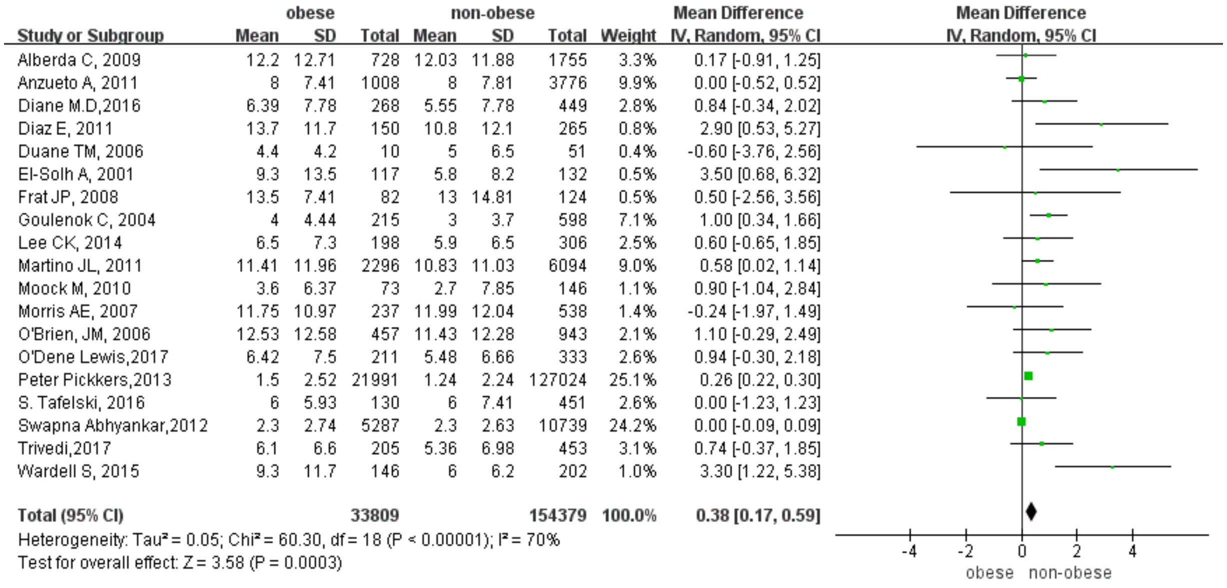


**C.ICU LOS of obese vs non-obese patients**

| Study or Subgroup | Experimental Mean | Experimental SD | Experimental Total | Control Mean | Control SD | Control Total | Weight | Mean Difference IV, Random, 95% CI |
|---|---|---|---|---|---|---|---|---|
| **1.1.1 obese vs normal** | | | | | | | | |
| Alberda C, 2009 | 11.77 | 12.14 | 557 | 11.7 | 11.7 | 937 | 4.0% | 0.07 [-1.19, 1.33] |
| Anzueto A, 2011 | 8 | 7.41 | 792 | 8 | 8.15 | 1995 | 11.6% | 0.00 [-0.63, 0.63] |
| Diane M.D,2016 | 6 | 5.92 | 216 | 5 | 4.44 | 200 | 5.8% | 1.00 [-0.00, 2.00] |
| Martino JL, 2011 | 11.5 | 11.63 | 1772 | 10.7 | 11.7 | 3490 | 10.8% | 0.80 [0.13, 1.47] |
| Morris AE, 2007 | 10.7 | 9.4 | 183 | 11.8 | 13.1 | 301 | 1.7% | -1.10 [-3.11, 0.91] |
| O'Brien, JM, 2006 | 11.9 | 11.1 | 326 | 11.6 | 12.7 | 544 | 2.5% | 0.30 [-1.31, 1.91] |
| Peter Pickkers,2013 | 1.55 | 2.5 | 18999 | 1.2 | 2.15 | 74883 | 32.4% | 0.35 [0.31, 0.39] |
| Swapna Abhyankar,2012 | 2.3 | 2.74 | 5287 | 2.3 | 2.67 | 5463 | 31.2% | 0.00 [-0.10, 0.10] |
| **Subtotal (95% CI)** | | | **28132** | | | **87813** | **100.0%** | **0.25 [-0.02, 0.52]** |
| Heterogeneity: Tau² = 0.06; Chi² = 46.20, df = 7 (P < 0.00001); I² = 85% | | | | | | | | |
| Test for overall effect: Z = 1.84 (P = 0.07) | | | | | | | | |
| **1.1.2 overweight vs normal** | | | | | | | | |
| Alberda C, 2009 | 12.4 | 12.07 | 818 | 11.7 | 11.7 | 937 | 1.2% | 0.70 [-0.42, 1.82] |
| Anzueto A, 2011 | 8 | 7.41 | 1781 | 8 | 8.15 | 1995 | 5.5% | 0.00 [-0.50, 0.50] |
| Diane M.D,2016 | 6 | 5.18 | 249 | 5 | 4.44 | 200 | 1.8% | 1.00 [0.11, 1.89] |
| Martino JL, 2011 | 11 | 10.07 | 2604 | 10.7 | 11.7 | 3490 | 4.6% | 0.30 [-0.25, 0.85] |
| Morris AE, 2007 | 13.5 | 13 | 237 | 11.8 | 13.1 | 301 | 0.3% | 1.70 [-0.52, 3.92] |
| Peter Pickkers,2013 | 1.3 | 2.37 | 54121 | 1.2 | 2.15 | 74883 | 48.2% | 0.10 [0.07, 0.13] |
| Swapna Abhyankar,2012 | 2.3 | 2.59 | 5276 | 2.3 | 2.65 | 5463 | 37.4% | 0.00 [-0.10, 0.10] |
| Trivedi,2017 | 4.9 | 5.3 | 194 | 5.7 | 8 | 259 | 1.0% | -0.80 [-2.03, 0.43] |
| **Subtotal (95% CI)** | | | **65280** | | | **87528** | **100.0%** | **0.09 [-0.04, 0.21]** |
| Heterogeneity: Tau² = 0.01; Chi² = 13.51, df = 7 (P = 0.06); I² = 48% | | | | | | | | |
| Test for overall effect: Z = 1.37 (P = 0.17) | | | | | | | | |
| **1.1.3 severe obesity vs normal** | | | | | | | | |
| Alberda C, 2009 | 13.6 | 14.37 | 171 | 11.7 | 11.7 | 937 | 8.7% | 1.90 [-0.38, 4.18] |
| Anzueto A, 2011 | 8 | 7.41 | 216 | 8 | 8.15 | 1995 | 20.0% | 0.00 [-1.05, 1.05] |
| Diane M.D,2016 | 8 | 6.66 | 52 | 5 | 4.44 | 200 | 11.1% | 3.00 [1.09, 4.91] |
| Martino JL, 2011 | 11.1 | 13.04 | 524 | 10.7 | 11.7 | 3490 | 18.4% | 0.40 [-0.78, 1.58] |
| Morris AE, 2007 | 11.75 | 10.97 | 54 | 11.8 | 13.1 | 301 | 5.0% | -0.05 [-3.33, 3.23] |
| O'Brien, JM, 2006 | 14.1 | 15.6 | 131 | 11.6 | 12.7 | 544 | 6.1% | 2.50 [-0.38, 5.38] |
| Peter Pickkers,2013 | 1.2 | 2.59 | 2992 | 1.2 | 2.15 | 74883 | 30.7% | 0.00 [-0.09, 0.09] |
| **Subtotal (95% CI)** | | | **4140** | | | **82350** | **100.0%** | **0.72 [-0.07, 1.52]** |
| Heterogeneity: Tau² = 0.54; Chi² = 15.36, df = 6 (P = 0.02); I² = 61% | | | | | | | | |
| Test for overall effect: Z = 1.78 (P = 0.08) | | | | | | | | |
| **1.1.4 underweight vs normal** | | | | | | | | |
| Alberda C, 2009 | 11.3 | 13.11 | 289 | 11.7 | 11.7 | 937 | 29.1% | -0.40 [-2.09, 1.29] |
| Anzueto A, 2011 | 8 | 8.89 | 184 | 8 | 8.15 | 199 | 28.2% | 0.00 [-1.71, 1.71] |
| Diane M.D,2016 | 5 | 5.92 | 18 | 5 | 4.44 | 200 | 10.5% | 0.00 [-2.80, 2.80] |
| Morris AE, 2007 | 10.8 | 11.1 | 50 | 11.8 | 13.1 | 301 | 7.1% | -1.00 [-4.41, 2.41] |
| Trivedi,2017 | 5.2 | 5 | 41 | 5.7 | 8 | 259 | 25.1% | -0.50 [-2.31, 1.31] |
| **Subtotal (95% CI)** | | | **582** | | | **1896** | **100.0%** | **-0.31 [-1.22, 0.60]** |
| Heterogeneity: Tau² = 0.00; Chi² = 0.38, df = 4 (P = 0.98); I² = 0% | | | | | | | | |
| Test for overall effect: Z = 0.67 (P = 0.50) | | | | | | | | |

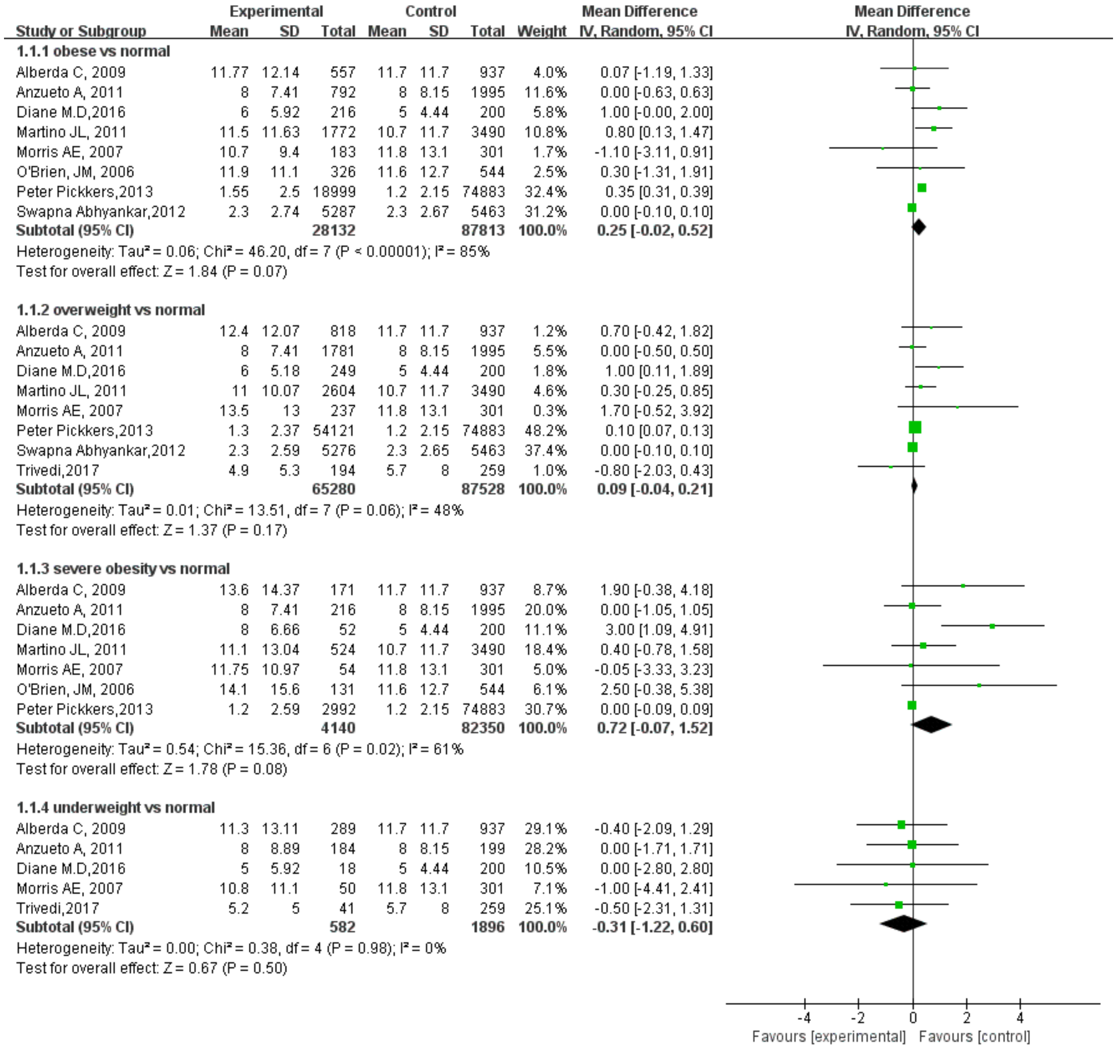


**D.ICU LOS of different BMI classification**

| Study or Subgroup | obese Mean | obese SD | obese Total | non-obese Mean | non-obese SD | non-obese Total | Weight | Mean Difference IV, Random, 95% CI |
|---|---|---|---|---|---|---|---|---|
| Alberda C, 2009 | 21.8 | 20.63 | 728 | 24.51 | 25.73 | 1755 | 6.4% | -2.71 [-4.63, -0.79] |
| Anzueto A, 2011 | 17 | 15.25 | 1008 | 17 | 16.3 | 3776 | 12.5% | 0.00 [-1.08, 1.08] |
| Diane M.D,2016 | 17.78 | 15.97 | 268 | 15.89 | 16.1 | 449 | 4.5% | 1.89 [-0.53, 4.31] |
| Diaz E, 2011 | 22.2 | 16.5 | 150 | 18.2 | 14.6 | 265 | 2.9% | 4.00 [0.83, 7.17] |
| Duane TM, 2006 | 9.8 | 8.5 | 10 | 8.1 | 7.1 | 51 | 1.0% | 1.70 [-3.92, 7.32] |
| Lee CK, 2014 | 14.7 | 14.6 | 198 | 14.9 | 18 | 306 | 3.4% | -0.20 [-3.06, 2.66] |
| Martino JL, 2011 | 23.52 | 24.72 | 2296 | 24.94 | 30.03 | 6094 | 10.7% | -1.42 [-2.68, -0.16] |
| Morris AE, 2007 | 21.46 | 25.08 | 237 | 20.46 | 17.31 | 538 | 2.4% | 1.00 [-2.51, 4.51] |
| O'Brien, JM, 2006 | 26.87 | 24.35 | 457 | 24.7 | 26.15 | 943 | 3.6% | 2.17 [-0.62, 4.96] |
| O'Dene Lewis,2017 | 12.78 | 15.65 | 211 | 11.1 | 10.33 | 333 | 4.6% | 1.68 [-0.71, 4.07] |
| Peter Pickkers,2013 | 11.73 | 12.59 | 21991 | 12 | 12.59 | 127024 | 21.9% | -0.27 [-0.45, -0.09] |
| Swapna Abhyankar,2012 | 8 | 6.67 | 5287 | 7.5 | 4.56 | 10739 | 21.8% | 0.50 [0.30, 0.70] |
| Trivedi,2017 | 13.6 | 12.6 | 205 | 14.9 | 18.68 | 453 | 4.4% | -1.30 [-3.74, 1.14] |
| **Total (95% CI)** | | | **33046** | | | **152726** | **100.0%** | **0.05 [-0.52, 0.63]** |

Heterogeneity: Tau² = 0.38; Chi² = 58.78, df = 12 (P < 0.00001); I² = 80%
Test for overall effect: Z = 0.19 (P = 0.85)

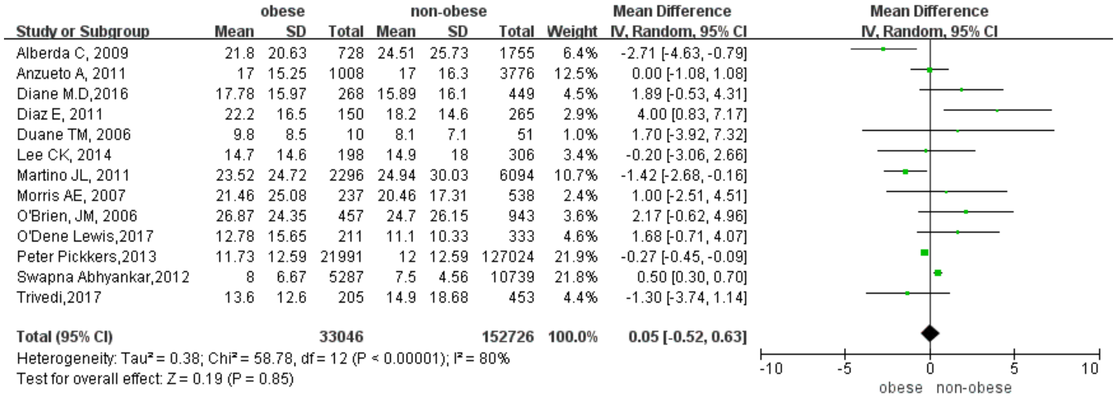


**E.hospital LOS of obese vs non-obese patients**

| Study or Subgroup | Experimental Mean | Experimental SD | Experimental Total | Control Mean | Control SD | Control Total | Weight | Mean Difference IV, Random, 95% CI |
|---|---|---|---|---|---|---|---|---|
| **1.1.1 obese vs normal** | | | | | | | | |
| Alberda C, 2009 | 24.32 | 21.6 | 557 | 24.6 | 24.96 | 937 | 15.1% | -0.28 [-2.68, 2.12] |
| Anzueto A, 2011 | 17 | 15.56 | 792 | 17 | 16.3 | 1995 | 28.1% | 0.00 [-1.30, 1.30] |
| Diane M.D,2016 | 17 | 14.8 | 216 | 14.5 | 14.8 | 200 | 12.0% | 2.50 [-0.35, 5.35] |
| Martino JL, 2011 | 24 | 24.52 | 1772 | 25.5 | 31.41 | 3490 | 24.5% | -1.50 [-3.05, 0.05] |
| Morris AE, 2007 | 19 | 17.1 | 183 | 19.4 | 17.2 | 301 | 10.3% | -0.40 [-3.55, 2.75] |
| O'Brien, JM, 2006 | 26.9 | 23.2 | 326 | 24.7 | 23.6 | 544 | 10.0% | 2.20 [-1.01, 5.41] |
| **Subtotal (95% CI)** | | | **3846** | | | **7467** | **100.0%** | **0.07 [-1.09, 1.22]** |
| Heterogeneity: Tau² = 0.80; Chi² = 8.41, df = 5 (P = 0.14); I² = 41% | | | | | | | | |
| Test for overall effect: Z = 0.12 (P = 0.91) | | | | | | | | |
| **1.1.2 overweight vs normal** | | | | | | | | |
| Alberda C, 2009 | 24.4 | 26.59 | 818 | 24.6 | 24.96 | 937 | 14.3% | -0.20 [-2.62, 2.22] |
| Anzueto A, 2011 | 17 | 16.3 | 1781 | 17 | 16.3 | 1995 | 31.8% | 0.00 [-1.04, 1.04] |
| Diane M.D,2016 | 17 | 17.02 | 249 | 14.5 | 14.8 | 200 | 10.8% | 2.50 [-0.45, 5.45] |
| Martino JL, 2011 | 24.2 | 28.07 | 2604 | 25.5 | 31.41 | 3490 | 24.5% | -1.30 [-2.80, 0.20] |
| Morris AE, 2007 | 21.8 | 17.4 | 237 | 19.4 | 17.2 | 237 | 9.9% | 2.40 [-0.71, 5.51] |
| Trivedi,2017 | 14.1 | 17.1 | 194 | 15.5 | 19.8 | 259 | 8.6% | -1.40 [-4.81, 2.01] |
| **Subtotal (95% CI)** | | | **5883** | | | **7118** | **100.0%** | **0.04 [-1.08, 1.16]** |
| Heterogeneity: Tau² = 0.74; Chi² = 8.53, df = 5 (P = 0.13); I² = 41% | | | | | | | | |
| Test for overall effect: Z = 0.07 (P = 0.94) | | | | | | | | |
| **1.1.3 severe obesity vs normal** | | | | | | | | |
| Alberda C, 2009 | 22 | 27.85 | 171 | 24.6 | 24.96 | 937 | 16.5% | -2.60 [-7.07, 1.87] |
| Anzueto A, 2011 | 17 | 14.07 | 792 | 17 | 16.3 | 1995 | 27.0% | 0.00 [-1.21, 1.21] |
| Diane M.D,2016 | 21 | 19.98 | 52 | 14.5 | 14.8 | 200 | 12.9% | 6.50 [0.70, 12.30] |
| Martino JL, 2011 | 21.9 | 25.33 | 524 | 25.5 | 31.41 | 3490 | 23.6% | -3.60 [-6.01, -1.19] |
| Morris AE, 2007 | 29.8 | 41.3 | 54 | 19.4 | 17.2 | 301 | 5.2% | 10.40 [-0.79, 21.59] |
| O'Brien, JM, 2006 | 26.8 | 27.1 | 131 | 24.7 | 23.6 | 544 | 14.8% | 2.10 [-2.95, 7.15] |
| **Subtotal (95% CI)** | | | **1724** | | | **7467** | **100.0%** | **0.41 [-2.40, 3.22]** |
| Heterogeneity: Tau² = 7.22; Chi² = 18.09, df = 5 (P = 0.003); I² = 72% | | | | | | | | |
| Test for overall effect: Z = 0.28 (P = 0.78) | | | | | | | | |
| **1.1.4 underweight vs normal** | | | | | | | | |
| Alberda C, 2009 | 23.1 | 26.67 | 289 | 24.6 | 24.96 | 937 | 27.4% | -1.50 [-4.97, 1.97] |
| Anzueto A, 2011 | 18 | 19.26 | 184 | 17 | 16.3 | 1995 | 39.8% | 1.00 [-1.87, 3.87] |
| Diane M.D,2016 | 16.5 | 14.06 | 18 | 14.5 | 14.8 | 200 | 7.1% | 2.00 [-4.81, 8.81] |
| Morris AE, 2007 | 18.2 | 15.3 | 50 | 19.4 | 17.2 | 301 | 15.1% | -1.20 [-5.86, 3.46] |
| Trivedi,2017 | 15.8 | 16.4 | 41 | 15.5 | 19.8 | 259 | 10.6% | 0.30 [-5.27, 5.87] |
| **Subtotal (95% CI)** | | | **582** | | | **3692** | **100.0%** | **-0.02 [-1.83, 1.79]** |
| Heterogeneity: Tau² = 0.00; Chi² = 1.78, df = 4 (P = 0.78); I² = 0% | | | | | | | | |
| Test for overall effect: Z = 0.02 (P = 0.98) | | | | | | | | |

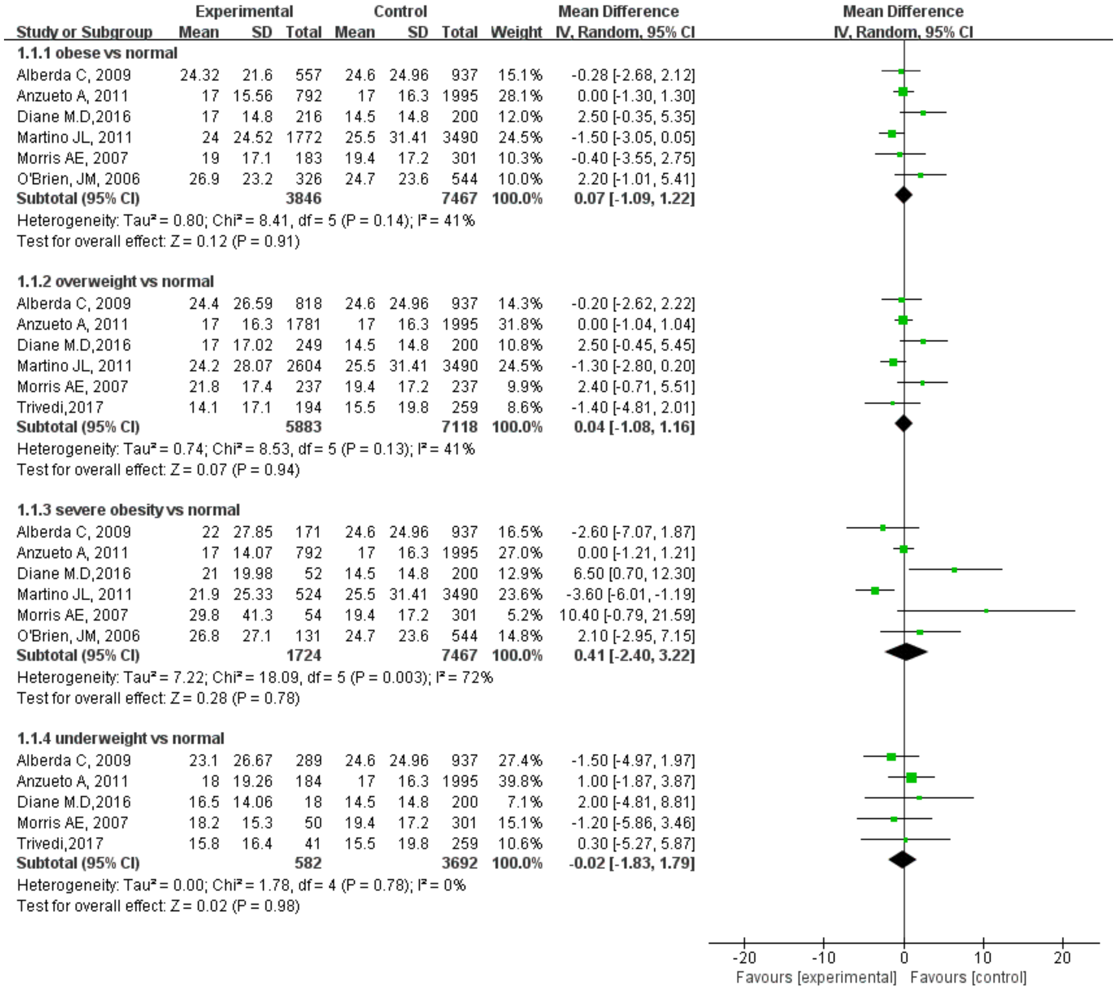


**F. hospital LOS of different BMI classification**
