# Supplementary material for: Is body mass index associated with outcomes of mechanically ventilated adult patients in intensive critical units? A systematic review and meta-analysis
Source: PLoS One. 2018 Jun 8;13(6):e0198669. doi: 10.1371/journal.pone.0198669 (PMC5993298; doi:10.1371/journal.pone.0198669)
Supplement: S1 Table — (PDF) [file pone.0198669.s004.pdf]

Newcastle-Ottawa Quality Assessment Scale for Cohort Studies

Note: A study can be awarded a maximum of one star for each numbered item within the Selection and Outcome categories. A maximum of two stars can be given for Comparability

Selection

1) Representativeness of the exposed cohort
   a) truly representative of the average cohort of patients with mechanical ventilation *
   b) somewhat representative of the average cohort of patients with mechanical ventilation*
   c) selected group of users eg nurses, volunteers
   d) no description of the derivation of the cohort

2) Selection of the non exposed cohort
   a) drawn from the same community as the exposed cohort *
   b) drawn from a different source
   c) no description of the derivation of the non exposed cohort

3) Ascertainment of exposure
   a) secure record (eg surgical records) *
   b) structured interview *
   c) written self report
   d) no description

4) Demonstration that outcome of interest was not present at start of study
   a) yes *
   b) no

Comparability

1) Comparability of cohorts on the basis of the design or analysis
   a) study controls for severity of illness *
   b) study controls for any additional factor * (This criteria could be modified to indicate specific control for a second important factor.)

Outcome

1) Assessment of outcome
   a) independent blind assessment *
   b) record linkage *
   c) self report
   d) no description

2) Was follow-up long enough for outcomes to occur
   a) yes (select an adequate follow up period for outcome of interest) *
   b) no

3) Adequacy of follow up of cohorts
   a) complete follow up - all subjects accounted for *
   b) subjects lost to follow up unlikely to introduce bias - small number lost - > 90 % follow up, or description provided of those lost) *
   c) follow up rate <90% (select an adequate %) and no description of those lost
   d) no statement

	A. Selection				B. Comparability of cohorts	C. Outcome		
	Represent-ativeness of exposed cohort	Selection of non-exposed	Ascertainment of Exposure	Outcome not present at start		Assessment of exposure	F/U long enough?	Adequacy of F/U
S. Tafelski, 2016	*	*	*	*	*		*	
Wardell S, 2015	*	*	*	*		*	*	*
Lee CK, 2014	*		*	*	*		*	*
O'Brien JM ,2012	*	*	*	*	*	*	*	*
Martino JL,2011	*	*	*			*	*	*
Anzueto A,2011	*	*	*		*		*	*
Díaz E, 2011	*		*	*		*	*	
Moock M, 2010	*	*	*	*	*	*		*
Alberda C, 2009	*	*	*	*		*	*	*
Frat JP, 2008	*		*			*	*	*
Morris AE, 2007	*	*	*	*	*		*	*
Peake SL, 2007	*		*			*	*	*
Duane TM, 2006	*		*	*			*	
O'Brien JM Jr,2006	*	*	*	*		*	*	*
Ray DE, 2005	*		*	*		*		*
Goulenok C, 2004	*		*			*	*	
El-Solh A, 2001	*			*	*		*	*
Brown CVR, 2005					*		*	*

	A. Selection				B. Comparability of cohorts	C. Outcome		
	Represent-ativeness of exposed cohort	Selection of non-exposed	Ascertainment of Exposure	Outcome not present at start		Assessment of exposure	F/U long enough?	Adequacy of F/U
Diane M.D2016			*	*	*		*	*
O’Dene Lewis2017		*	*	*	*	*	*	*
Trivedi2017		*	*	*	*	*	*	*
Peter Pickkers2013		*	*	*	*	*	*	*
Swapna Abhyankar2012	*	*	*	*	*	*	*	*
